# Estrogen Repression of MicroRNAs Is Associated with High Guanine Content in the Terminal Loop Sequences of Their Precursors

**DOI:** 10.3390/biomedicines5030047

**Published:** 2017-08-14

**Authors:** Amit Cohen, Mario Alberto Burgos-Aceves, Tamar Kahan, Yoav Smith

**Affiliations:** 1 Genomic Data Analysis Unit, The Hebrew University of Jerusalem-Hadassah Medical School, P.O. Box 12272, Jerusalem 91120, Israel; amitcohen879@gmail.com (A.C.); yoavs@ekmd.huji.ac.il (Y.S.); 2 Laboratorio de Endocrinología, Centro de Investigaciones Biológicas del Noroeste, Instituto Politécnico Nacional 195, Playa Palo de Santa Rita Sur, La Paz 23096, B.C.S., Mexico; 3 Bioinformatics Unit, The Hebrew University of Jerusalem-Hadassah Medical School, P.O. Box 12272, Jerusalem 91120, Israel; tamark@ekmd.huji.ac.il

**Keywords:** estrogen, microRNA, Terminal loop, Guanine, cigarette smoke, xenoestrogens, DNA adducts, cancer

## Abstract

Widespread microRNA (miRNA) repression is a phenomenon observed in mammals after exposure to cigarette smoke and in many types of cancer. A comprehensive reduction in miRNA expression after treatment with the hormone estrogen has also previously been described. Here, we reveal a conserved association of miRNA downregulation after estrogen exposure in zebrafish, mouse, and human breast cancer cell line, with a high guanine content in the terminal loop sequences of their precursors, and offer a possible link between estrogen-related miRNA-adducts formation and carcinogenesis. We also show common gene expression patterns shared by breast cancer tumors and estrogen-treated zebrafish, suggesting that this organism can be used as a powerful model system for the study of human breast cancer.

## 1. Introduction

Estrogens, the female reproductive sex hormones, have wide-ranging physiological effects throughout a woman’s lifetime and are also implicated in the development and progression of several diseases, including cancer [1,2]. The potential carcinogenic activity of estrogen can occur through nuclear estrogen receptor (ER)-mediated and/or nongenomic signaling pathways [3], and may also be associated with global repression of microRNAs (miRNAs, miRs), a group of small noncoding RNAs with a pivotal role in the regulation of gene expression and function [4,5]. Several studies have shown that exposure to the hormone estrogen leads to widespread downregulation in miRNA expression [6,7,8], and miRNAs deregulation was also found to be implicated in estrogen-related mammary and endometrial cancer [9]. These alterations can occur as a result of altering the transcription of miRNA genes, as was shown in the case of miRNA regulation by c-Myc [10], suppression of miRNA export from the nucleus [11], or at any stage of the miRNA maturation process [12].

Another potential carcinogenic activity of estrogen involves the oxidative metabolism of estrone (E1) or 17β-estradiol (E2) to catechol estrogens, and the reactive quinone metabolites [13] that bind covalently with purines in DNA to form specific DNA adducts at the *N*-3 of adenine (Ade) and *N*-7 of guanine (Gua) [14]. These adducts generate apurinic sites that can be converted into mutations by error-prone repair, which in turn may initiate tumorigenesis [15].

The effects of RNA adduction have often been considered less important than the effects of DNA adduction, despite their similar structure and the possibility of similar reactivity, because unlike genomic DNA, modifications to RNA do not lead to mutations. Therefore, the number of reports on RNA adduct analysis are significantly fewer [16]. Izzotti and Pulliero [17] evaluated the formation of guanine-adducts (Gua-adducts) in miRNAs of the lungs of mice that were exposed to cigarette smoke (CS) and found that the Gua-adducts level was about 6-fold higher than that detectable in the DNA of the same animals, concluding therefore that miRNAs are more sensitive than DNA to the formation of adducts induced by exposure to CS [17]. They also showed, using bioinformatic analysis, that the Gua content of the terminal loop (TL) of miRNAs that are involved in stress response is higher than the Gua content of the other miRNAs, and suggested that environmental carcinogens form miRNA adducts that can modify the structure of precursor miRNAs (pre-miRNAs), thus blocking their access to the catalytic site of Dicer and alter miRNAs expression. Some findings indicate that mutagens form stable complexes with Dicer, therefore competing with the natural pre-miRNAs substrates for Dicer binding, resulting in a reduction in the amount of mature miRNAs [18]. Chemopreventive agents, such as isothiocyanates and indoles—Phenethyl isothiocyanate (PEITC) and Indole-3-carbinol (I3C), respectively—display affinity for Dicer, may compete with mutagens for Dicer binding, and can reverse the observed reduction in miRNA maturation after CS exposure [18,19]. We have previously suggested that this effect of PEITC and I3C on miRNA expression after CS exposure can be due to their known anti-estrogenic properties, which may potentially attenuate the effects of elevated levels of estrogen metabolites inside the lungs as a result of CS exposure [20,21], and therefore may also reduce the formation of estrogen–nucleic acid adducts.

Despite the growing body of work indicating that estrogen-induced DNA damage is an important factor for carcinogenesis, relatively little is known about the mechanism by which estrogens initiate the carcinogenic process [22]. We show here—using miRNA expression data analysis of zebrafish liver, mouse uterus and human MCF-7 cells—the conserved association of estrogen-related miRNA repression with a high Gua content in the terminal loop sequences of their precursors, and suggest a new mechanism for the formation of estrogen–Gua miRNA adducts and carcinogenesis.

## 2. Materials and Methods

### 2.1. Estrogen-Treated Zebrafish Dataset

Microarray gene expression data of male zebrafish livers after E2 treatment (published in Cohen and Smith [8]) was retrieved from the Gene Expression Omnibus (GEO) database under the accession number GSE45562 (https://www.ncbi.nlm.nih.gov/geo/query/acc.cgi?acc=GSE45562).

### 2.2. Breast Cancer Dataset

Microarray gene expression data (published in Harrell et al. [23]) of normal human breast tissue (12 samples) and breast cancer tumors (181 samples) was retrieved from the GEO database under the accession number GSE26338 (https://www.ncbi.nlm.nih.gov/geo/query/acc.cgi?acc=GSE26338).

### 2.3. Bioinformatic Tools

All miRNA precursor sequences were obtained from the Sanger Institute miRBase database version 21 (http://microrna.sanger.ac.uk/sequences/). GOrilla (http://cbl-gorilla.cs.technion.ac.il/), DAVID v6 functional annotation tool (http://david.abcc.ncifcrf.gov/), and the STRING database v10 (http://string-db.org/), were used to identify enriched GO terms of genes and their interaction networks. Gene expression analysis of microarray data was performed using Partek-Genomics-Suite v6.6 software (Partek Inc., Chesterfield, MO, USA).

### 2.4. Nucleotide Composition Analysis

Calculation of nucleotide composition in miRNA precursors was determined using the compseq algorithm (http://emboss.bioinformatics.nl/cgi-bin/emboss/compseq). Input sequences included pre-miRNA stem-loops and terminal loops of E2-repressed miRNAs in zebrafish liver ([8]; ArrayExpress database accession number E-MEXP-3866; http://www.ebi.ac.uk/arrayexpress/experiments/E-MEXP-3866/), mouse uterus ([7]; GEO database accession number GSE13858; https://www.ncbi.nlm.nih.gov/geo/query/acc.cgi?acc=GSE13858) and human breast cancer MCF-7 cells ([6]; GEO database accession number GSE17460; https://www.ncbi.nlm.nih.gov/geo/query/acc.cgi?acc=GSE17460) (miRNA lists and their sequences are presented in Tables S1 and S2). All known pre-miRNA sequences producing the specific mature E2-repressed miRNAs were selected for the analysis. As controls, 50 miRNA sequences of zebrafish, mouse and human, were randomly selected and used in each matching calculation.

### 2.5. Statistical Data Analysis

Normalized gene expression microarrays data were analyzed using Partek-Genomics-Suite v6.6 software. For the data from the zebrafish estrogen treatment experiment [8], one-way ANOVA was performed with the false discovery rate (FDR) < 0.05. A *t*-test was performed on the gene expression data for breast cancer vs. normal breast [23].

## 3. Results and Discussion

**Figure 1:**
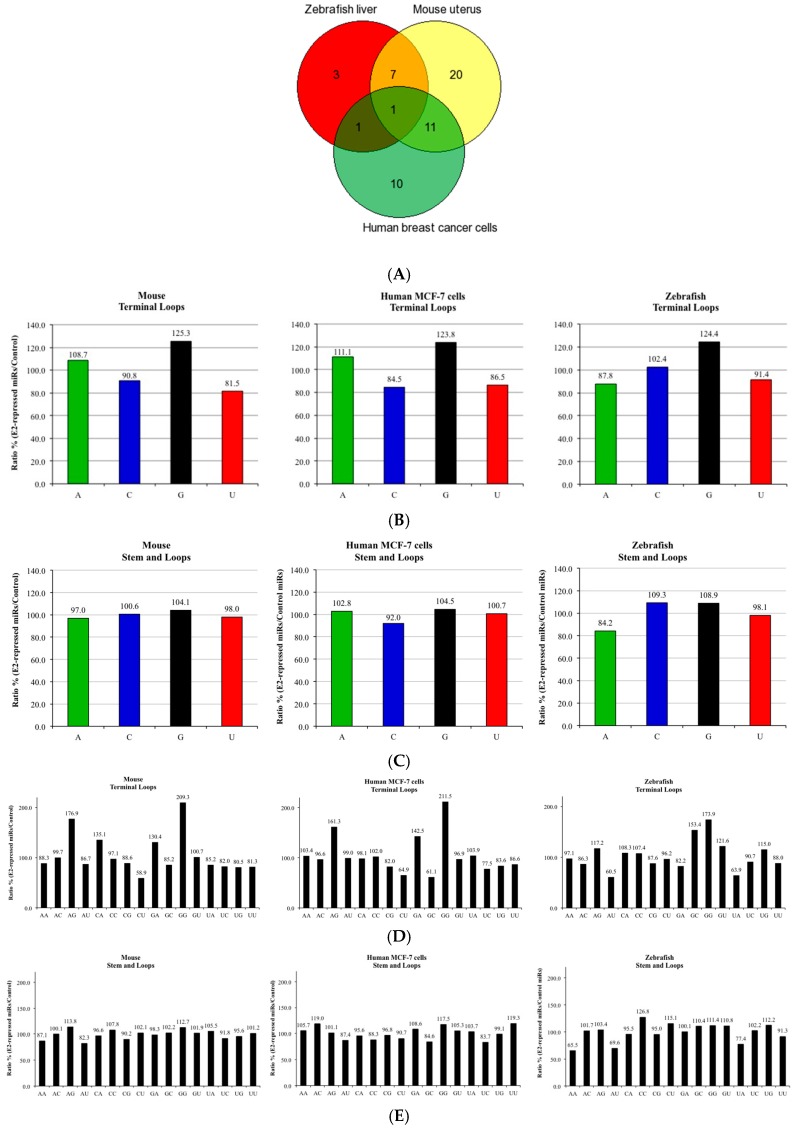

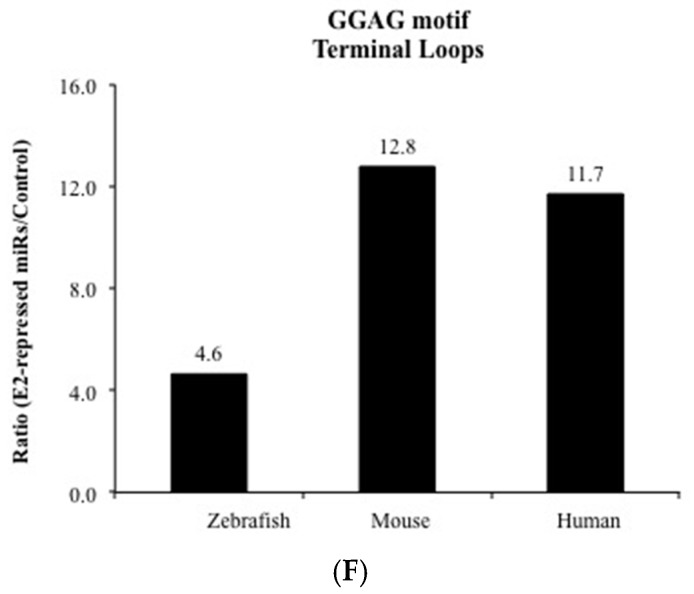
Nucleotide composition of E2-repressed miRNAs. (**A**) Venn diagram showing the overlap between E2-repressed miRNAs of E2-treated zebrafish liver, mouse uterus, and human breast cancer MCF-7 cells; (**B**) Single nucleotide composition of terminal loops of the precursors of E2-repressed zebrafish, mouse, and human miRNAs; (**C**) Single nucleotide composition of stem-loops of E2-repressed zebrafish, mouse, and human miRNAs; (**D**) Dual nucleotide composition of terminal loops of the precursors of E2-repressed zebrafish, mouse, and human miRNAs; (**E**) Dual nucleotide composition of stem-loops of E2-repressed zebrafish, mouse, and human miRNAs; (**F**) Relative enrichment of GGAG motif in terminal loops of the precursors of E2-repressed zebrafish, mouse, and human miRNAs.

Since widespread downregulation of miRNA expression happens after both CS and estrogen exposures [20], and is also known to be a common phenomenon in many types of human cancer [5], we raised the hypothesis that both CS and estrogen exposure form miRNA adducts that cause repression of tumor suppressor miRNAs and induction of their target oncogenes, which may ultimately lead to carcinogenesis. To test this hypothesis, we conducted the same bioinformatics analysis done by Izzotti and Pulliero [17] on miRNAs involved in stress response using our previously published results that demonstrated a reduction in miRNA expression after estrogen exposure in adult male zebrafish livers [8] and the results of other studies showing widespread repression of miRNAs after estrogen treatment in the uterus of ovariectomized female mice and human breast cancer cells [6,7]. Lists of the most significant downregulated miRNAs, as were obtained by microarray experiments after estrogen treatment, were selected for bioinformatic analysis (Table S1). As shown in Figure 1A, an overlap was observed between the lists of E2-repressed miRNAs of the different experimental models, where miR-26a was included in all three of them (Table S1). For each of these E2-repressed miRNAs, and for the 50 other randomly selected miRNAs that were used as a control, the complete stem-loop pre-miRNA sequences and the precursor terminal loop sequences were analyzed for evaluation of nucleotide composition (Table S2).

The results show that the zebrafish, mouse, and human estrogen-downregulated miRNAs have, respectively, a 24.4%, 25.3%, and 23.8% higher G content in the terminal loop sequences than in the control miRNA group (Figure 1B). In contrast, when the same analysis was conducted with the complete pre-miRNA sequences, no such difference was revealed (8.9%, 4.1% and 4.5%, in zebrafish, mouse, and human, respectively) (Figure 1C). Izzotti and Pulliero show in their results a remarkable increase in the dual GUA (GG) content of the terminal loop sequences of miRNAs involved in stress response [17]. Our results show that the same enrichment is also observed in the aforementioned zebrafish, mouse, and human estrogen-downregulated miRNAs, which have, respectively, a 73.9%, 109.3%, and 111.5%, higher GG content in the terminal loop sequences than in the control miRNA group (Figure 1D). Also in this case, when the same analysis was conducted with the complete pre-miRNA sequences, no such a difference was revealed (11.4%, 12.7% and 17.5%, in zebrafish, mouse, and human, respectively) (Figure 1E). Together, the above results show that Gua content enrichment is predominantly observed in the precursor terminal loop sequences of the estrogen-downregulated miRNAs.

The miRNA terminal loop is an important platform for different RNA binding proteins that act as activators or repressors of Drosha and Dicer processing [24]. Such examples are miRNAs with the tetra-nucleotide sequence motif GGAG in their terminal loop, that are regulated through binding of the RNA binding protein Lin28 [25] and miRNAs with the sequence AGGGU in the terminal loop, which are regulated by KH-type splicing regulatory protein (KSRP) [26,27]. Motif analysis revealed a high enrichment for the sequence motif GGAG in the terminal loop sequences of the zebrafish, mouse, and human estrogen-downregulated miRNAs, relative to the control miRNA group (Figure 1F). This enrichment was also observed in the analysis when solely considering the miRNA families (Figure S1). Furthermore, eight of the E2-repressed miRNAs also contained the AGGGU motif in their terminal loop (let-7 family members, miR-26a and miR-125a), whereas this motif does not exist in any of the control miRNA terminal loop sequences (Table S2).

Intriguingly, several of the E2-repressed miRNAs were also shown to function as tumor suppressors. For example, miRNAs of the let-7 family repress the expression of known oncogenes, including k-Ras and c-Myc [28,29]. MiR-143 and miR-145 are co-expressed miRNAs that function as tumor suppressors and their repression by k-Ras potentiates the oncogenic k-Ras signaling by a feed-forward loop [30,31]. Interestingly, miR-145 also participates in a regulatory loop involving the tumor suppressor p53 and targets ER-alpha in human breast cancer cells [32], and the processing of the primary miRNAs (pri-miRNAs) of miR-143 and miR-145 by Drosha was also shown to be regulated in a p53-dependent manner [33]. The E2-regulated miR-30c has been reported to be a tumor suppressor in endometrial cancer [34], miR-107 functions as a tumor-suppressor gene in head and neck squamous cell carcinoma and was shown to mediate p53 tumor-suppressor function in human colon cancer cells [35,36], and miR-26a strongly inhibited estrogen-stimulated breast cancer cells and tumor growth [6,37].

**Figure 2:**
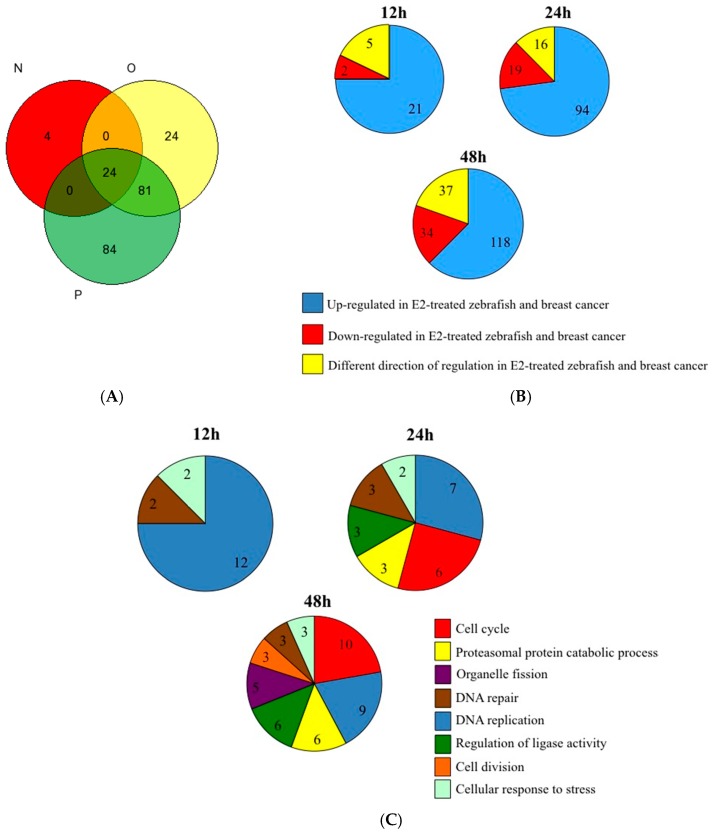

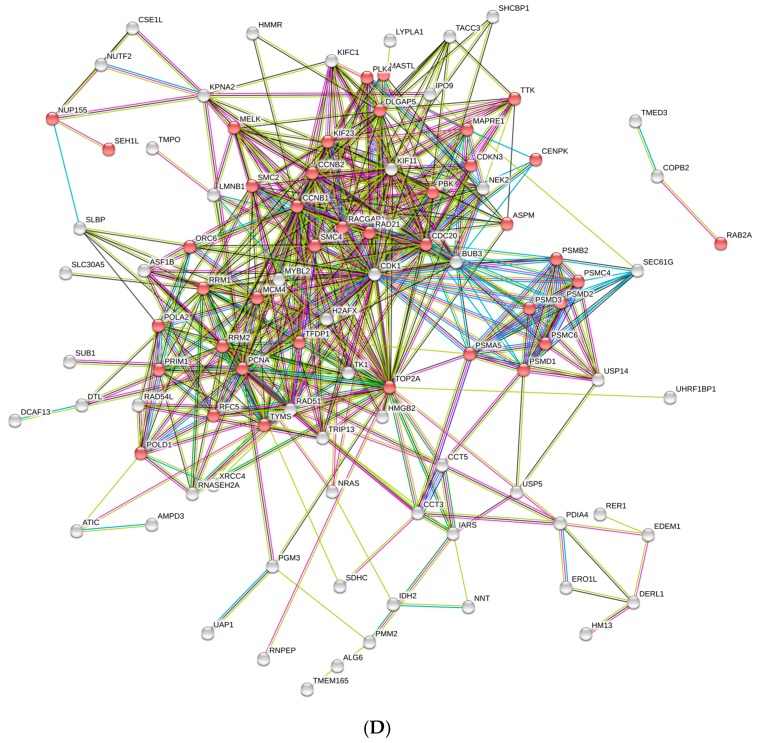
Comparison of gene expression profiles between E2-treated zebrafish and human breast cancer. (**A**) Venn diagram showing the overlap between differentially expressed genes of E2-treated zebrafish and human breast cancer at different time points after E2 treatment: 12 h (N), 24 h (O), 48 h (P); (**B**) Direction of regulation of common differentially expressed genes of zebrafish at different time points after E2 treatment and of human breast cancer. Graphs represent the number of regulated genes; (**C**) Enriched gene ontology (GO) terms of common differentially-expressed genes to E2-treated zebrafish and human breast cancer, as were identified using the DAVID functional annotation tool. Shown are significant −log2 Benjamini *p*-values of the biological process terms of differentially expressed genes at 12, 24 and 48 h; (**D**) Gene network of the common up-regulated genes to 48 h E2-treated zebrafish and human breast cancer. Presented is the most enriched “mitotic cell cycle” biological process GO term GO: 0000278 (41 out of 118 common upregulated genes), as was identified and visualized by the STRING database. The 41 common upregulated mitotic cell cycle genes are marked with red circles.

It is widely accepted practice to use the zebrafish as a model organism for different studies on human health risks. However, in relation to cancer research, relatively little is known about the similarities at the molecular level between zebrafish and human tumors [38]. Here, we conducted gene expression analysis using data obtained from normal human breast tissue and breast cancer tumors [23], and compared them to gene expression profiles that were received after estrogen treatment in the adult zebrafish liver [8]. A total of 217 common genes have been changed in human breast cancer tumors and also differentially expressed at different time points after estrogen exposure in the zebrafish (Figure 2A, Table S3). The results indicate that most of the common genes show the same direction of expression pattern (82%, 87%, and 80%, at 12, 24 and 48 h after estrogen treatment, respectively), and frequently tend to be induced in breast cancer and upregulated after estrogen treatment (75%, 73%, and 62%, at 12, 24 and 48 h after estrogen treatment, respectively) (Figure 2B). Looking for biological process enrichment in the list of common genes for all three time points revealed that DNA replication is the most significant (*p* = 1.48^−9^, using the Gorilla tool). DNA replication was also one of the most significant biological processes at each separate time point (*p* = 1.87^−13^, 5.07^−8^ and 1^−9^ for 12, 24, and 48 h respectively, using the DAVID tool). The cell cycle biological process was significant only during later time points (*p* = 3.24^−7^ and 1.65^−11^ for 24 and 48 h respectively) (Figure 2C), whereas the “mitotic cell cycle” was the most enriched biological process GO term (*p* = 1.15^−24^) at 48 h, as was identified by the STRING database (Figure 2D). Of the 41 common upregulated mitotic cell cycle genes (Figure 2D), seven genes (*CCNB1*, *CDC20*, *MELK*, *MYBL2*, *ORC6L*, *RRM2*, *TYMS*) belong to the *PAM50* gene set used to classify breast cancer subtypes.

Taken together, our results show that many of the genes which are common to human breast cancer and E2-treated zebrafish are related to DNA replication and mitosis; processes that are found to be frequently misregulated in different cancers [39]. These results are supported by the study of Lam et al. [40] who compared the estrogen-responsive genes of zebrafish with genes of estrogen-exposed human breast cancer cell lines and found molecular conservation of estrogen-responsiveness, mainly in signaling pathways involved in cell cycle progression and DNA damage and repair.

In teleost fish, as in other oviparous animals, the hormone estrogen plays a key role in the regulation of the vitellogenesis process within the liver, where elevated serum levels of E2 induce the expression of vitellogenin genes and eventually result in the formation of yolky eggs inside the ovary [41]. We have previously described significant differences in miRNA expression profiles between the livers of vitellogenic and non-vitellogenic zebrafish females [8]. Noticeably, several of the estrogen-repressed miRNAs (miR-26, miR-107, miR-126 and miR-145) were also reduced by the physiological estrogen levels of vitellogenic females [8]. Thus, it can be assumed that estrogen-miRNA adducts could also affect fish reproduction and other estrogen-regulated biological processes [42], and could be used as bioindicators of xenoestrogens that pollute our environment [43].

## 4. Conclusions

**Figure 3:**
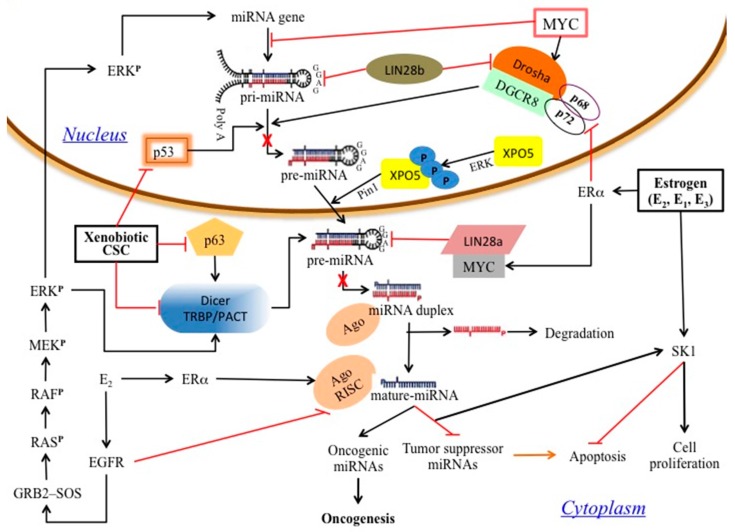
A model summarizing the findings revealed during this study and the potential disruption of miRNA processing events (denoted by the red crosses), leading to carcinogenesis. Arrows represent activation and blocked arrows indicate repression. CSC: cigarette-smoke condensate; EGFR: EGF receptor; ER: estrogen receptor; ERK: extracellular signal-regulated kinase; GRB: growth factor receptor-bound protein; SOS: Son of Sevenless; SK1: Sphingosine kinase 1; XPO5: Exportin-5.

The results of this study suggest that the formation of estrogen–nucleic acid adducts can potentially be a mechanism by which miRNA expression is altered by estrogens which can eventually lead to carcinogenesis (Figure 3). It will be interesting to identify whether a high terminal loop Gua content is also associated with the widespread downregulation of miRNA that was clinically observed in many types of human cancers [5,44,45]. It is noteworthy that single nucleotide polymorphisms (SNPs) which involve A > G and G > A nucleotide transitions, and are located at the terminal loop sequences of pre-miRNAs, were shown to be associated with the development of breast, gastric and kidney cancers [46,47,48,49].

Since cellular RNA is under constant attack by various environmental agents that damage the molecule, elucidating the association between exposure to xenostrogens and miRNA-adducts formation may also help gain a better understanding of the health implications of these endocrine disrupting chemicals for both humans and wildlife [50,51].

## Supplementary Materials

The following are available online at www.mdpi.com/2227-9059/5/3/47/s1.
