# Supplementary material for: Estrogen Repression of MicroRNAs Is Associated with High Guanine Content in the Terminal Loop Sequences of Their Precursors
Source: Biomedicines. 2017 Aug 14;5(3):47. doi: 10.3390/biomedicines5030047 (PMC5618305; doi:10.3390/biomedicines5030047)
Supplement: Supplementary file 1 [file biomedicines-05-00047-s001.pdf]

Supplementary Materials: Estrogen Repression of MicroRNAs is Associated with High Guanine Content in the Terminal Loop Sequences of Their Precursors

Amit Cohen, Mario Alberto Burgos-Aceves, Tamar Kahan and Yoav Smith

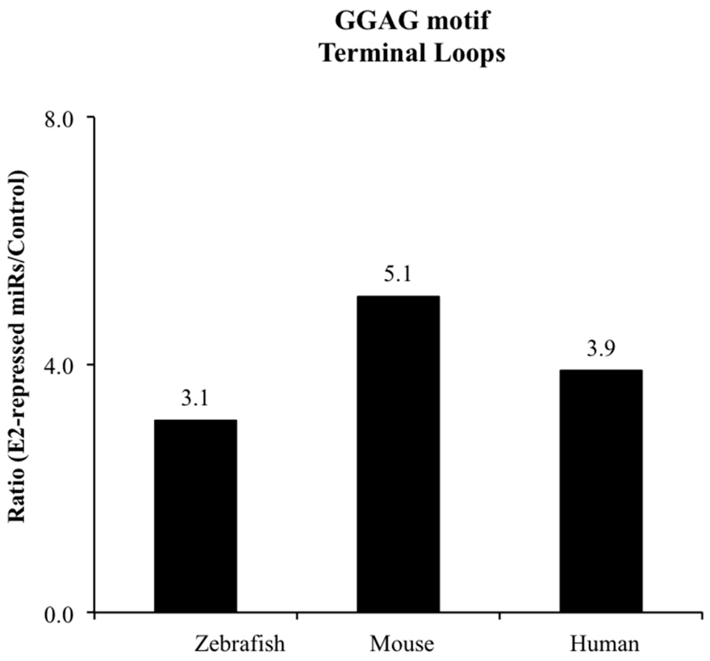

Figure S1. Relative enrichment of GGAG motif in terminal loops of the precursors of E2-repressed zebrafish, mouse and human miRNAs. The relative enrichment is represented as miRNA families.

Table S1. Lists of E2-repressed miRNAs in zebrafish liver, mouse uterus and human breast cancer MCF-7 cells and the overlap between them.

	**Species**			**Common to**				**Only in**		
	**Zebrafish**	**Mouse**	**Human**	**Zebrafish-Mouse-Human**	**Zebrafish-Mouse**	**Zebrafish-Human**	**Mouse- Human**	**Zebrafish**	**Mouse**	**Human**
	107a MI0001963	let-7d MI0000405	let-7a-1 MI0000060	miR-26a	let-7d	miR-26b	let-7g	miR-101b	miR-133a	miR-181a
	let-7d-1 MI0001868	26a-1 MI0000573	let-7a-2 MI0000061		miR-199		let-7f	miR-107a	miR-16	miR-181b
	let-7d-2 MI0001870	26a-2 MI0000706	let-7a-3 MI0000062		miR-143		let-7c	miR-338	miR-100	miR-181d
	143-1 MI0002007	125b-1 MI0000725	let-7c MI0000064		miR-126a		let-7a		miR-184	miR-193a
	143-2 MI0002008	125b-2 MI0000152	let-7f-1 MI0000067		miR-125b		miR-27b		miR-191	miR-193b
	125a-1 MI0001972	126a MI0000153	let-7f-2 MI0000068		miR-125a		miR-27a		miR-194	miR-203a
	125a-2 MI0001973	143 MI0000257	let-7g MI0000433		miR-145		miR-24		miR-195a	miR-203b
	125b-1 MI0001975	145a MI0000169	21 MI0000077				miR-23b		miR-214	miR-499a
	125b-2 MI0001976	199a-1 MI0000241	23a MI0000079				miR-23a		miR-29a	miR-499b
**microRNA**	125b-3 MI0001977	199a-2 MI0000713	23b MI0000439				miR-21		miR-30a	miR-520d*
	145 MI0002010	let-7a-1 MI0000556	24-1 MI0000080				miR-200c		miR-30c	
	126a MI0001979	let-7a-2 MI0000557	24-2 MI0000081						miR-30d	
	26a-1 MI0001923	let-7b MI0000558	26a-1 MI0000083						miR-30e	
	26a-2 MI0001925	let-7c-1 MI0000559	26a-2 MI0000750						miR-320	
	26a-3 MI0001926	let-7c-2 MI0000560	26b MI0000084						miR-497	
	26b MI0001927	let-7e MI0000561	27a MI0000085						miR-99a	
	199-1 MI0001373	let-7f-1 MI0000562	27b MI0000440						miR-99b	
	199-2 MI0001374	let-7f-2 MI0000563	98 MI0000100						let-7b	
	199-3 MI0001375	let-7g MI0000137	181a-1 MI0000289						let-7e	

101b MI0001961	let-7i MI0000138	181a-2 MI0000269	let-7i
338-1 MI0002063	16-1 MI0000565	181b-1 MI0000270	
338-2 MI0002064	16-2 MI0000566	181b-2 MI0000683	
	21a MI0000569	181d MI0003139	
	23a MI0000571	193a MI0000487	
	23b MI0000141	193b MI0003137	
	24-1 MI0000231	200a MI0000737	
	24-2 MI0000572	200c MI0000650	
	27a MI0000578	203a MI0000283	
	27b MI0000142	203b MI0017343	
	29a MI0000576	499a MI0003183	
	30a MI0000144	499b MI0017396	
	30c-1 MI0000547	520d* MI0003164	
	30c-2 MI0000548		
	30d MI0000549		
	30e MI0000259		
	99a MI0000146		
	99b MI0000147		
	100 MI0000692		
	125a MI0000151		
	133a-1 MI0000159		
	133a-2 MI0000820		
	184 MI0000226		
	191 MI0000233		
	194-1 MI0000236		
	194-2 MI0000733		
	195a MI0000237		
	200c MI0000694		
	214 MI0000698		
	320 MI0000704		

497a MI0004636

E2: 17beta-estradiol; miRNA: microRNA; MCF-7: Michigan Cancer Foundation-7.

Table S2. Sequences of stem-loops and terminal loops of E2-repressed and control miRNAs in zebrafish liver, mouse uterus and human breast cancer MCF-7 cells. The motif AGGGU is underlined in terminal loop sequences.

microRNA	Stem-Loop	Terminal loop
	Zebrafish-Estrogen regulated miRNAs (Cohen and Smith, 2014)	
>dre-mir-107a MI0001963	UCUGUGUGCUCUGAGCUUCUUUACAGUGUUGUCUUGUGGCAUGGAGAUCAAGCAGCAUUGUACAGGGCUAUCACAGCACACUGAACAGC	UGGCAUGGAGAUCA
>dre-let-7d-1 MI0001868	UGUGCGUUGCGGUGUGAGGUAGUUGGUUGUAUGGUUUUGCAUAAUAAACAGCCCGGAGUUAACUGUACAACCUUCUAGCUUUCCCUGCGGCUGCACG	UUGCAUAAUAAACAGCCCGGAGUUAA
>dre-let-7d-2 MI0001870	CGCUGCAGGCUGAGGUAGUUGGUUGUAUGGUUUUGCAUCAUAAUCAGCCUGGAGUUAACUGUACAACCUUCUAGCUUUCCCUGCGGUG	UUGCAUCAUAAUCAGCCUGGAGUUAA
>dre-mir-143-1 MI0002007	GAUCUACAGUCGUCUGGCCCGCGGUGCAGUGCUGCAUCUCUGGUCAACUGGGAGUCUGAGAUGAAGCACUGUAGCUCGGGAGGACAACACUGUCAGCUC	GGUCAACUGGGAGUC
>dre-mir-143-2 MI0002008	GAUCUACAGUCGUCUGGCCCGCGGUGCAGUGCUGCAUCUCUGGUCAACUGGGAGUCUGAGAUGAAGCACUGUAGCUCGGGAGGACAACACUGUCAGCUC	GGUCAACUGGGAGUC
>dre-mir-125a-1 MI0001972	GUAUGUCUCUUUGUCCCUGAGACCCUUAACCUGUGAGGUCAAACUAGGUCACAGGUGAGGUCCUCAGGAACAGGGCUGCAUGC	AGGUCAAACUAGGUCACAGGU
>dre-mir-125a-2 MI0001973	GAUCAGUCCAAAUCGAUGUAUGUCUGUGUCCCUGAGACCCUUAACCUGUGAUGUCUUCCAAGGUCACAGGUGAGGUCCUUGGGAACACGGCUGUAUAUGAUGACGUC	AUGUCUUCCAAGGU
>dre-mir-125b-1 MI0001975	UUCUGUUGCAGGUUGGCGGUUGGUCUGCAAAUGUGCCUCUCACAAUCCCUGAGACCCUAACUUGUGACGUUUUCCUGUUAUGUGCACGGGUUAGGUUCUUGGGAGCUGAGAGGGGUGCUCUGUCAUCAGCCCGCCGGCGUCGGAA	CGUUUUCCUGUUAUGUGC
>dre-mir-125b-2 MI0001976	GUGCCCCUCUCCUUCCCUGAGACCCUAACUUGUGACGUUCUGCUUCGAUGUCCACGGGUUGGGUUCUCGGGAGCUGUGAGAGGCAC	CGUUCUGCUUCGAUGUCCA
>dre-mir-125b-3 MI0001977	CCCGUGCGGCCACCGCUGCACUCCUCCUGGUCCCUGAGACCCUAACUUGUGAGCUUUGUGUGCUAAAAAUCACAGGUUAAGCUCUUGGGACCUGGGCAGAGGGCAAAAGCACUGG	GCUUUGUGUGCUAAAAAUC
>dre-mir-145 MI0002010	UCAGUCUUCAUCAUUUCCUCAUCCCCGGGGUCCAGUUUUCCCAGGAAUCCCUUGGGCAAUCGAAAGGGGGAUUCCUGGAAAUACUGUUCUUGGGGUUGGGGGUGGACUACUGA	UUGGGCAAUCGAAAGGG

>dre-mir-126a MI0001979	GAGCCAUUUUAACUGCUUCACAGUCCAUUAUUACUUUUGGUACGCGCUAGGCCAGACUCAAACUCGUACCGUGAGUAAUAAUGCACUGUGGCAGUGGGUUU	CUAGGCCAGACUCAAAC
>dre-mir-26a-1 MI0001923	UUUGGCCUGGUUCAAGUAAUCCAGGAUAGGCUUGUGAUGUCCGGAAAGCCUAUUCGGGAUGACUUGGUUCAGGAAUGA	UGUGAUGUCCGGAAAG
>dre-mir-26a-2 MI0001925	GUGUGGACUUGAGUGCUGGAAGUGGUUGUUCCCUUGUUCAAGUAAUCCAGGAUAGGCUGUCUGUCCUGGAGGCCUAUUCAUGAUUACUUGCACUAGGUGGCAGCCGUUGCCCUUCAUGGAACUCAUGC	GUCUGUCCUGGAGG
>dre-mir-26a-3 MI0001926	CUAAGCUGAUACUGAGUCAGUGUGUGGCUGCAACCUGGUUCAAGUAAUCCAGGAUAGGCUUUGUGGACUAGGGUUGGCCUGUUCUUGGUUACUUGCACUGGGUUGCAGCUACUAAACAACUAAGAAGAUCAGAAGAG	UUGUGGACUAGGGUU
>dre-mir-26b MI0001927	GCAUUUGGCCUUUGCCUGGUUCAAGUAAUCCAGGAUAGGUUAGUUCCCACUAGUACGGCCUAUUCUUGGUUACUUGUUUCAGGAGGAGGCUACGAGC	AGUUCCCACU
>dre-mir-199-1 MI0001373	UCCUGCUCCGUCAUCCCAGUGUUCAGACUACCUGUUCAGGAUCAUACUGGUGUACAGUAGUCUGCACAUUGGUUAGACUGUGCAUGG	AGGAUCAUACUGGUG
>dre-mir-199-2 MI0001374	GGAGUUUUUGUGGACGCCCGUCCCGCCUGCCCAGUGUUCAGACUACCUGUUCAGGAAUUAGUGUUUGUACAGUAGUCUGCACAUUGGUUAGGCUGG	AGGAAUUAGUGUUUG
>dre-mir-199-3 MI0001375	CCUCCCCCUCGCCUGCCCAGUGUUCAGACUACCUGUUCAUCAUGCUGCAGCUGAACAGUAGUCCGCACAUUGGUUAGGCUGGGCUGGGACACACACAC	AUCAUGCUGCAGCUGA
>dre-mir-101b MI0001961	GCUCCUCCGUCAUGAAUUGUCCAUUUUCAGUUAUCAUGGUACCGGUGCUGUGUGCCUGUCAAGUACAGUACUAUGAUAACUGAAGAUUGACGGUGCCAAACAUCAGUGGAGU	GUGUGCCUGUCAAG
>dre-mir-338-1 MI0002063	GGUUUCUCCCUGCAACAAUCUCCUGAUGCUGCCUGAGUGUUUUUCUUCCACUCCAGCAUCAGUGAUUUUGUUGCCGGAGGUCACC	GUGUUUUUCUUCCAC
>dre-mir-338-2 MI0002064	GUGUGUGUGUGUGUUUCUGGUGCCUGCUGAGAACAAUAUCCUGAUGCUGAAUGAGUGUGUUGAAGGAAACUCCAGCAUCAGUGAUUUUGUUGCCAGAGGAGCACUUUGGGCAUCCUGUGUAUAU	GUGUGUUGAAGGAAAC
	Zebrafish-Control miRNAs	
>dre-mir-10a MI0001363	UGUCUGUCAUCUAUAUAUACCCUGUAGAUCCGAAUUUGUGUGAAUAUACAGUCGCAAAUUCGUGUCUUGGGGAAUAUGUAGUUGACAUAAACACAACGC	GUGAAUAUACAGUCG
>dre-mir-9-1 MI0001880	GGGGUUGGCUGUUAUCUUUGGUUAUCUAGCUGUAUGAGUGUUAUUCAUUCUUCAUAAAGCUAGAUAACCGAAAGUAACAAGAAUCCC	GUGUUAUUCAUUCUUCA
>dre-mir-24-1 MI0001921	ACCUGAGCUCCGGUGCCUUCUGAGCUGAUAUCAGUUGUAGUAAAUCACUGGCUCAGUUCAGCAGGAACAGGAGUGUGGCC	GUUGUAGUAAAUCAC
>dre-mir-27a MI0001928	UCUGGAUAUGAUGUCUGCUGAAGUUUCGUGAGGUGCAGGACUUAGCUCACUCUGUGAACAGAUCUCGGAUAUCCUAUGUUCACAGUGGCUAAGUUCCGCUCCUCUGAGGCCCACACUCGAAAUCAGCCAGG	GAUCUCGGAUAUCCUAUG
>dre-mir-34b MI0003690	GGGGUUGGUCUGUAGGCAGUGUUGUUAGCUGAUUGUUUCAUAUGAACUAUAAUCACUAACCAUACUGCCAACACAACAACCUACA	UUUCAUAUGAACUA
>dre-mir-92a-1 MI0001951	UGGUCCCUUUCUGCGCAGGUUGGGAUUGGUAGCAAUGCUGUGUGUUUUGAAGGUAUUGCACUUGUCCCGGCCUGUAAAGGAUUGU	GUGUGUUUUGAAGG

dre-mir-365-2 MI0002069	AGGCAGCAAGAAAAAUGAGGGACUUUUAGGGGCAGCUGUGUUUUAUUAACCCAGUCAUAAUGCCCCUAAAAAUCCUUAUUGCUCUUGCAAUUUUCAGC	GCUGUGUUUUAUUAACCCAGUCA
>dre-mir-430a-7 MI0002116	GUCACUAUCGGUACCCUCACAAAGGCACUGACUUGGAUGCUGUAAUUGGUAAGUGCUAUUUGUUGGGGUAGUUUCAAGUGAC	UGGAUGCUGUAAUUGG
>dre-mir-181c MI0002024	GGGUCCUGAUUCACAUUCAUUGCUGUCGGUGGGUUUUAUCUCUUCGACUCGCCGGACAAUGAAUGAGAACUACGGCUC	UUUUAUCUCUUCGA
>dre-mir-187-1 MI0001370	UGACCUGUGGCUGGGCCAGGGGCUGCAACACAGGACAUGGGAGCUGUCUCUCACUCCCGCUCGUGUCUUGUGUUGCAGCCAGUGGAACG	GGGAGCUGUCUCUCACUCCCGCU
>dre-mir-19c MI0001905	UGGAAAAGCCCUGUUAUCUGGGGUGAGUUUUGCAGGAUUGCAUCCGGCUUUAUUACAACAUGCUGUGCAAAUCCAUGCAAAACUCGCUGCGCCAGGGACAAACCA	CUUUAUUACAACAUGC
>dre-mir-23a-1 MI0001913	GCUGUGGCGGGGAGGGUUCCUGGCACCGUGAUUUGGUGGAUAAACAGAAAUGAAAAUCACAUUGCCAGGGAUUUCCACUCCUGCACGGU	UUGGUGGAUAAACAGAAAUGAAA
>dre-mir-153a MI0002021	GGUUGCCAGUGUCAUUUUUGUGAUGUUGCAGCUAGUUAUAUGAGCCCAGUUGCAUAGUCACAAAAGUGAUCAUUGGAAACU	AGUUAUAUGAGCCCAG
>dre-mir-7147 MI0023606	GAGUGCCCAGUGCUGUACCAUGCUGGUAGCCAGUAUGAAAUAGGGCUUGCUGGUAACCAGCGUUGUGCCCCACUGGUUGCUC	AUGAAAUAGGGCUU
>dre-mir-2197 MI0010857	AGUGUAAAAGGAAAACGUUGUGAUGAUUCGACUCAUAUGGUGCUAUGCAGUAAUAGAGGGCUGGAAGCAUUUUGCUUGAGGAGAGUCUAAUCACUAGCAGGUACACAUUG	CUAUGCAGUAAUAGAGGGCUGGAAG
>dre-mir-124-6 MI0001971	GGGUGGUGACACAGGCCCGCCACUCUGCGUGUUCACGGCGGACCUUGAUUUAAUAUCCAUACAAUUAAGGCACGCGGUGAAUGCCAAGAGAGGGGUCUUAAAACGACAAACCC	UUAAUAUCCAUACAAU
>dre-mir-219-2 MI0001385	UGAUUGUCCAAACGCAAUUCUUGUGAAAUGUCGAGCGAUCAGUCGAGAAUUGUGCCUGGACAUCUGUUGCUGGAGGCUCC	GUGAAAUGUCGAGCGAUCAGUC
>dre-mir-2198 MI0010847	CGGCGAGCAAUACUCAUAACUCCCAAGCGCCUGCGAACGCAAGCUCUCCUUACCCCGGUGCAGAUGGAGCUCGUGUCCCAAGGCGCCUCCAGGCCUAAACCCGGGCCUGC	CUCCUUACCCCGGUGCAGAUGG
>dre-mir-202 MI0002040	CUGUUCCUUUUUCCUAUGCAUAUACCUCUUUGACAUGCUGCUUUAAAGAGGCAUAGGGCAUGGGAAAAUGGGGCGG	ACAUGCUGCUUUAA
>dre-mir-216a MI0001382	GCUGAUUUUUGGCAUAAUCUCAGCUGGCAACUGUGAGUAGUGUUUUCAUCCCUCUCACAGGCGCUGCUGGGGUUCUGUCACACACAGCA	GUAGUGUUUUCAUCCCUC
>dre-mir-301b MI0002061	AAGGUCUGUUGCUUUGACGAUGUUGCACUACUGAACCAUCUAAUCAAGCAGUGCAAUAGUAUUGUCAUUGCAUUCGGCUUU	UGAACCAUCUAAUCAAG
>dre-mir-1306 MI0023609	UCCACCACCUCCCCUGCAAACGUCCAGUGACGCAGAGGAAAUGGACGUUAGCUCUGGUGGUGAUGGACA	GUGACGCAGAGGAAA
>dre-mir-217 MI0001383	AUGAGAACUUUCUGAUGUUGGUGAUACUGCAUCAGGAACUGAUUGGAUGAUAUUCAGGAGCCAUCAGUUCCUGAUGCACUCCCAUCAGCAUCGAAAGA	AUGAUAUUCAGGAG
>dre-mir-20b MI0001899	GAGUUUGUCCUGGCAGUUCCAAAGUGCUCACAGUGCAGGUAGUGCCAGUGGAUCUACUGCAAUGUCUGCACUUCAAGUAUUGCCGGACGCCUUC	UGCCAGUGGAUCU

>dre-mir-29b-2 MI0001934	UCUUCCUCCAGAUGCUGGUUUCACAUGGUGGUUUAGAUGUGUUCUACCAAAGUCUAGCACCAUUUGAAAUCAGUGUUCUUGGGGAGGG	GAUGUGUUCUACCAAAGUC
>dre-mir-93 MI0001954	GUGUGUGUUAAAAGUGCUGUUUGUGCAGGUAGUGUGUUUCCUCUACUGUAGGAGCAGCACUUCACAACACACAC	GUGUGUUUCCUC
>dre-mir-190a MI0002027	UCUGGAGGUGAGGUAGACCUGGAAGCCUUUCUGCAGGCCUCUGUUUGAUAUGUUUGAUAUAUUAGGUUGUUAUUCUGUCCAACUAUAUAUCAAACAUAUUCCUACAGUGUCCUGCUCUGUCUCCAG	UGUUAUUCUGUCCA
>dre-mir-196a-1 MI0001372	CGCGCGGCUGGUGCGUGGUUUAGGUAGUUUCAUGUUGUUGGGAUUGGCUUCCUGGCUCGACAACAAGAAACUGCCUUGAUUACGUCAGUUCGUCUUCAUCAAGGGC	AUUGGCUUCCUGG
>dre-mir-375-1 MI0002072	UGCACUUGCUUUACGUUGAGCCACACGCACAAUACAUGUGGAUUCAGUUUUGUUCGUUCGGCUCGCGUUAAGCAAGUGCA	ACAUGUGGAUUCAGU
>dre-mir-222a MI0001388	GCCGGUGGCUUGUUCGGGUGCUCAUGAGAUGCUCAGUAGUCAGUGUAGAUCCUGUGUCACAAUCAGCAGCUACAUCUGGCUACUGGGUCUCUGAUGGCAUUUUCUGCU	UGUGUCACAAUCA
>dre-mir-499 MI0004766	ACUGAGAGGGAGGCAGUUAAGACUUGCAGUGAUGUUUAGAGAAAUGUCACAUGAACAUCACUUUAAGUCUGUGCUGGCUCCUGUUCUGAGU	GAGAAAUGUCACAUG
>dre-mir-205 MI0001378	AAACUACUGUGCAUUCUAUCCUUCAUUCCACCGGAGUCUGUGUAGUUGUUCAAUCAGAUUUCAGUGGUGUGAAGUGUAGGAAACACGGA	UGUAGUUGUUCAAUCA
>dre-mir-140 MI0002003	GUGUUUGUCUCCUGUGUCCCGUCAGUGGUUUUACCCUAUGGUAGGUUACGUCAUGCUGUUCUACCACAGGGUAGAACCACGGACGGGAUGUCUGGAGGUGUCUGC	GUUACGUCAUGCUGUUC
>dre-mir-1788 MI0010838	UCAACCACUGUCUUGUAUCCGAGGCUUGUUUUAAGUUGCCUGCGAUCUCUUAAUGACUCAGGCAGCUAAAGCAAGUCUGGGAGGCCAGAGACAACACGACA	AUCUCUUAAUGACU
>dre-mir-363 MI0002067	UAAAUGCAAAUAUUUGUCUUGCUGUUUUCGGGUGGAUGACUCUGCAAUUUUAUUAGUGAUGGAAAAACUUCAAUAAAAAUUGCACGGUAUCCAUCUGUAAUCCGCUGGAUUCCAUUACCUGCGUUUG	AUUAGUGAUGGAAAAACUUCAAUAAA
>dre-mir-182 MI0001368	GUAUUUGGCAAUGGUAGAACUCACACUGGUGAGGUAGUCAGAUCCGGUGGUUCUAGACUUGCCAACUA	CUGGUGAGGUAGUCAGAUCCGG
>dre-mir-17a-1 MI0001897	GGACUUUCUUGAGUGGACUUGGUUGGUGUCAAUGUAUUGUCAAAGUGCUUACAGUGCAGGUAGUAUUAUGGAAUAUCUACUGCAGUGGAGGCACUUCUAGCAAUACACUUGACCAUUUUAACCUUCCUCCAGGCAUCC	GUAUUAUGGAAUAUCU
>dre-mir-727 MI0004771	CUGUAUGUCAUUUUCAGUCUUCAAUUCCUCCCAGCCCGUACCCAUCGAAACUGUGAGUUGAGGCGAGUUGAAGACUUAAAGUGCUGUACAG	CCGUACCCAUCGAAACUGUGA
>dre-mir-2188 MI0010850	GGACAAUAUCAGCAAUGUGAGAAAGGUCCAACCUCACAUGUCCUGUGAGGCUGAAGGAAGGCUGUGUGAGGUUAGACCUAUCCCACACGGCCCGUAUUCUUCCC	UGUGAGGCUGAAGGAAGG
>dre-mir-204-2 MI0002042	GUGUUGCUCUAGUGACCAGUUUGUGACCUCCUGGGUUUCCCUUUGUCAUCCUAUGCCUGCAGUUCCUGAUGAGGCUGGGACAGCAAAGGGAGGUUCAGAUGUCGACCUGUACUACAGUCAAUAC	GCAGUUCCUGAUGAG
>dre-mir-206-1 MI0002045	GAAUGUUGCCUCUUGUGAAGACAUGCUUCCUUAUAUGCCCAUAUUAAUGCUCAAGUUAUGGAAUGUAAGGAAGUGUGUGGUUUCAGGGGGAAAUUUGUC	UUAAUGCUCAAGUUA
>dre-mir-193a-1 MI0002029	GACAUUAAUGUGUUAGAGGUUGGGUCUUUGCGGGCAAGGUGAGUAGUUAAAUUUACUCUCAACUGGCCUACAAAGUCCCAGUUUCUGGCUCAUGUUAUC	GUAGUUAAAUUUACUCUC

>dre-mir-137-2 MI0002001	GUUCUGUUGUUUCCCUCUAUAAAGGACUCUCUUCGGUGACGGGUAUUCUUGGGUGGAUAAUACGGCUCUC GUUGUUAUUGCUUAAGAAUACGCGUAGUCGAGGAGAGUCAUGUCGGCGGCGAGAGAGC	CGGCUCUCGUUG
>dre-mir-135b MI0003364	CUCUUUGCCCCGUAUGGCUUUUUAUUCCUAUCUGAGAAUUGCUCAGGACUCAUAUAGGGAUGGAAGCCAU GCAGGGCUGGGG	AGAAUUGCUCAGGACUC
>dre-mir-132-1 MI0001989	GUCUCCAUGGCGACCGUGGCAUUAGAUUGUUACUGUAGGAACAGAAUUUUUGGUAACAGUCUACAGCCAU GGUCGCUAGUGGGCA	GUAGGAACAGAAUUUUUGG
>dre-mir-133a-1 MI0001993	CAUCAAACCACAAUGCUUUGCUAAAGCUGGUAAAAUGGAACCAAAUCACCUCUUCAAUGGAUUUGGUCCC CUUCAACCAGCUGUAGCUAUGCUUUGAUG	CACCUCUUCAAUGGA
>dre-mir-203a MI0001376	GUGUUUGGGUCUCUUCUGGUCCCUCUGGUGCAGUGGUUCUUAACAGUUCAACAGUUCUAUCUCAAAAUUG UGAAAUGUUUAGGACCACUUGACCAG	UCUAUCUCAAAAUU
>dre-mir-723 MI0004767	UGCGUAGAGAUAAAGACAGUUUUAAAUGAUGUUACUUUUUUUCAAAUGGAGAAAGACAUCAAUUAAAUC UGUGCUUAUCUCUACAAGCA	UUUUCAAAUGGAGA
>dre-mir-221 MI0001387	GUCGUGAACCUGGCAUACAAUGUAGAUUUCUGUGUGGUACUAUCUACAGCUACAUUGUCUGCUGGGUUUC AGGCCAGCAGAAUAAUUCUGCUC	GUGGUACUAUCUAC
>dre-mir-138 MI0002002	UGUGUGCUGCAGCUGGUGUUGUGAAUCAGGCCGAUGUCACACGUCAGCGAUAACCCGGCUAUUUCACAAC ACCAGGGUGGCACCACA	GAUGUCACACGUCAGCGAUAACCCG
	Mouse-Estrogen regulated miRNAs (Yamagata et al. 2009)	
>mmu-let-7d MI0000405	AAUGGGUUCCUAGGAAGAGGUAGUAGGUUGCAUAGUUUUAGGGCAGAGAUUUUGCCCACAAGGAGUUAA CUAUACGACCUGCUGCCUUUCUUAGGGCCUUAUU	UUAGGGCAGAGAUUUUGCCCACAAGGAGUUAA
>mmu-mir-26a-1 MI0000573	AAGGCCGUGGCCUCGUUCAAGUAAUCCAGGAUAGGCUGUGCAGGUCCCAAGGGGCCUAUUCUUGGUUACU UGCACGGGGACGCGGGCCUG	GUGCAGGUCCCAAGGGG
>mmu-mir-26a-2 MI0000706	GGCUGCGGCUGGAUUCAAGUAAUCCAGGAUAGGCUGUGUCCGUCCAUGAGGCCUGUUCUUGAUUACUUGU UUCUGGAGGCAGCG	GUGUCCGUCCAUGAGG
>mmu-mir-125b-1 MI0000725	UGCGCUCCCCUCAGUCCCUGAGACCCUAACUUGUGAUGUUUACCGUUUAAAUCCACGGGUUAGGCUCUUG GGAGCUG	UGUUUACCGUUUAAAUCC
>mmu-mir-125b-2 MI0000152	GCCUAGUCCCUGAGACCCUAACUUGUGAGGUAUUUUAGUAACAUCACAAGUCAGGUUCUUGGGACCUAGG C	GGUAUUUUAGUAACAUC
>mmu-mir-126a MI0000153	UGACAGCACAUUAUUACUUUUGGUACGCGCUGUGACACUUCAAACUCGUACCGUGAGUAAUAAUGCGCGG UCA	CUGUGACACUUCAAAC

>mmu-mir-143 MI0000257	CCUGAGGUGCAGUGCUGCAUCUCUGGUCAGUUGGGAGUCUGAGAUGAAGCACUGUAGCUCAGG	UCAGUUGGGAGUC
>mmu-mir-145a MI0000169	CUCACGGUCCAGUUUUCCCAGGAAUCCCUUGGAUGCUAAGAUGGGGAUUCCUGGAAAUACUGUUCUUGAG	UGGAUGCUAAGAUGGGG
>mmu-mir-199a-1 MI0000241	GCCAUCCCAGUGUUCAGACUACCUGUUCAGGAGGCUGGGACAUGUACAGUAGUCUGCACAUUGGUUAGGC	AGGAGGCUGGGACAUGU
>mmu-mir-199a-2 MI0000713	UGGAAGCUUCAGGAGAUCCUGCUCCGUCGCCCCAGUGUUCAGACUACCUGUUCAGGACAAUGCCGUUGUA CAGUAGUCUGCACAUUGGUUAGACUGGGCAAGGGCCAGCA	AGGACAAUGCCGUUGU
>mmu-let-7a-1 MI0000556	UUCACUGUGGGAUGAGGUAGUAGGUUGUAUAGUUUUAGGGUCACACCCACCACUGGGAGAUAACUAUACA AUCUACUGUCUUUCCUAAGGUGAU	UUAGGGUCACACCCACC ACUGGGAGAUAA
>mmu-let-7a-2 MI0000557	CUGCAUGUUCCCAGGUUGAGGUAGUAGGUUGUAUAGUUUAGAGUUACAUCAAGGGAGAUAACUGUACAG CCUCCUAGCUUUCCUUGGGACUUGCAC	UAGAGUUACAUCAAGGG AGAUAA
>mmu-let-7b MI0000558	GCAGGGUGAGGUAGUAGGUUGUGUGGUUUCAGGGCAGUGAUGUUGCCCCUCCGAAGAUAACUAUACAACC UACUGCCUUCCCUGA	UCAGGGCAGUGAUGUUG CCCCUCCGAAGAUAA
>mmu-let-7c-1 MI0000559	UGUGUGCAUCCGGGUUGAGGUAGUAGGUUGUAUGGUUUAGAGUUACACCCUGGGAGUUAACUGUACAACC UUCUAGCUUUCCUUGGAGCACACU	UAGAGUUACACCCUGGG AGUUAA
>mmu-let-7c-2 MI0000560	ACGGCCUUUGGGGUGAGGUAGUAGGUUGUAUGGUUUUGGGCUCUGCCCCGCUCUGCGGUAACUAUACAAU CUACUGUCUUUCCUGAAGUGGCCGC	UUGGGCUCUGCCCCGCU CUGCGGUAA
>mmu-let-7e MI0000561	CGCGCCCCCCGGGCUGAGGUAGGAGGUUGUAUAGUUGAGGAAGACACCCGAGGAGAUCACUAUACGGCCU CCUAGCUUUCCCCAGGCUGCGCC	GAGGAAGACACCCGAGG AGAUCA
>mmu-let-7f-1 MI0000562	AUCAGAGUGAGGUAGUAGAUUGUAUAGUUGUGGGGUAGUGAUUUUACCCUGUUUAGGAGAUAACUAUAC AAUCUAUUGCCUUCCCUGAG	GUGGGGUAGUGAUUUUA CCCUGUUUAGGAGAUAA
>mmu-let-7f-2 MI0000563	UGUGGGAUGAGGUAGUAGAUUGUAUAGUUUUAGGGUCAUACCCCAUCUUGGAGAUAACUAUACAGUCUA CUGUCUUUCCCACG	UUAGGGUCAUACCCCAU CUUGGAGAUAA
>mmu-let-7g MI0000137	CCAGGCUGAGGUAGUAGUUUGUACAGUUUGAGGGUCUAUGAUACCACCCGGUACAGGAGAUAACUGUACA GGCCACUGCCUUGCCAGG	UGAGGGUCUAUGAUACC ACCCGGUACAGGAGAUA
>mmu-let-7i MI0000138	CUGGCUGAGGUAGUAGUUUGUGCUGUUGGUCGGGUUGUGACAUUGCCCGCUGUGGAGAUAACUGCGCAAG CUACUGCCUUGCUAG	GGUCGGGUUGUGACAUU GCCCGCUGUGGAGAUAA
>mmu-mir-16-1 MI0000565	AUGUCAGCGGUGCCUUAGCAGCACGUAAAUAUUGGCGUUAAGAUUCUGAAAUUACCUCCAGUAUUGACUG UGCUGCUGAAGUAAGGUUGGCAA	UUAAGAUUCUGAAAUUA CCU
>mmu-mir-16-2 MI0000566	CAUGCUUGUUCCACUCUAGCAGCACGUAAAUAUUGGCGUAGUGAAAUAAAUAUUAAACACCAAUAUUAUU GUGCUGCUUUAGUGUGACAGGGAUA	UAGUGAAAUAAAUAUUA AAC

>mmu-mir-21a MI0000569	UGUACCACCUUGUCGGAUAGCUUAUCAGACUGAUGUUGACUGUUGAAUCUCAUGGCAACAGCAGUCGAUGGGCUGUCUGACAUUUUGGUAUC	CUGUUGAAUCUCAUGG
>mmu-mir-23a MI0000571	CGGACGGCUGGGGUUCCUGGGGAUGGGAUUUGAUGCCAGUCACAAAUCACAUUGCCAGGGAUUUCCAACUGACCC	GAUGCCAGUCACAA
>mmu-mir-23b MI0000141	GGCUGCUUGGGUUCCUGGCAUGCUGAUUUGUGACUUGAGAUUAAAAUCACAUUGCCAGGGAUUACCACGCAACC	GUGACUUGAGAUUAAA
>mmu-mir-24-1 MI0000231	CUCCGGUGCCUACUGAGCUGAUAUCAGUUCUCAUUUCACACACUGGCUCAGUUCAGCAGGAACAGGAG	UCUCAUUUCACACAC
>mmu-mir-24-2 MI0000572	GCCUCUCUCCGGGCUCCGCCUCCCGUGCCUACUGAGCUGAAACAGUUGAUUCCAGUGCACUGGCUCAGUUCAGCAGGAACAGGAGUCCAGCCCCCUAGGAGCUGGCA	UGAUUCCAGUGCAC
>mmu-mir-27a MI0000578	UGGCCUGAGGAGCAGGGCUUAGCUGCUUGUGAGCAAGGUCCACAGCAAAGUCGUGUUCACAGUGGCUAAGUUCCGCCCCCUGGACCC	AGGUCCACAGCAAAGUCGUG
>mmu-mir-27b MI0000142	AGGUGCAGAGCUUAGCUGAUUGGUGAACAGUGAUUGGUUUCCGCUUUGUUCACAGUGGCUAAGUUCUGCACCU	AGUGAUUGGUUUCCGCUUUG
>mmu-mir-29a MI0000576	ACCCCUUAGAGGAUGACUGAUUUCUUUUGGUGUUCAGAGUCAAUAGAAUUUUCUAGCACCAUCUGAAAUCGGUUAUAAUGAUUGGGGA	AGUCAAUAGAAUUUUC
>mmu-mir-30a MI0000144	GCGACUGUAAACAUCCUCGACUGGAAGCUGUGAAGCCACAAAUGGGCUUUCAGUCGGAUGUUUGCAGCUGC	CUGUGAAGCCACAAAUGGG
>mmu-mir-30c-1 MI0000547	ACCAUGUUGUAGUGUGUGUAAACAUCCUACACUCUCAGCUGUGAGCUCAAGGUGGCUGGGAGAGGGUUGUUUACUCCUUCUGCCAUGGA	UGUGAGCUCAAGGUGG
>mmu-mir-30c-2 MI0000548	GAGUGACAGAUAUUGUAAACAUCCUACACUCUCAGCUGUGAAAAGUAAGAAAGCUGGGAGAAGGCUGUUUACUCUCUCUGCCUU	UGUGAAAAGUAAGAAAG
>mmu-mir-30d MI0000549	AAGUCUGUGUCUGUAAACAUCCCCGACUGGAAGCUGUAAGCCACAGCCAAGCUUUCAGUCAGAUGUUUGCUGCUACUGGCUC	CUGUAAGCCACAGCCAAG

>mmu-mir-30e MI0000259	GGGCAGUCUUUGCUACUGUAAACAUCCUUGACUGGAAGCUGUAAGGUGUUGAGAGGAGCUUUCAGUCGGAUGUUUACAGCGGCAGGCUGCCA	CUGUAAGGUGUUGAGAGGAG
>mmu-mir-99a MI0000146	CAUAAACCCGUAGAUCCGAUCUUGUGGUGAAGUGGACCGCGCAAGCUCGUUUCUAUGGGUCUGUG	GUGAAGUGGACCGCG
>mmu-mir-99b MI0000147	GGCACCCACCCGUAGAACCGACCUUGCGGGGCCUUCGCCGCACACAAGCUCGUGUCUGUGGGUCCGUGUC	GGGCCUUCGCCGCACA
>mmu-mir-100 MI0000692	CCUGUUGCCACAAACCCGUAGAUCCGAACUUGUGCUGAUUCUGCACACAAGCUUGUGUCUAUAGGUAUGUGUCUGUUAGG	CUGAUUCUGCACAC
>mmu-mir-125a MI0000151	CUGGGUCCCUGAGACCCUUUAACCUGUGAGGACGUCCAGGGUCACAGGUGAGGUUCUUGGGAGCCUGG	GGACGUCCAGGGUC
>mmu-mir-133a-1 MI0000159	GCUAAAGCUGGUAAAAUGGAACCAAAUCGCCUCUUCAAUGGAUUUGGUCCCCUUCAACCAGCUGUAGC	CGCCUCUUCAAUGGA
>mmu-mir-133a-2 MI0000820	AGAAGCCAAAUGCUUUGCUGAAGCUGGUAAAAUGGAACCAAAUCAGCUGUUGGAUGGAUUUGGUCCCCUUCAACCAGCUGUAGCUGCGCAUUGAUCACGCCGCA	CAGCUGUUGGAUGGA
>mmu-mir-184 MI0000226	CCUUUCCUUAUCACUUUUCCAGCCAGCUUUGUGACUCUAAGUGUUGGACGGAGAACUGAUAAGGGUAGG	UUUGUGACUCUAAGUGU
>mmu-mir-191 MI0000233	AGCGGGCAACGGAAUCCCAAAAGCAGCUGUUGUCUCCAGAGCAUUCCAGCUGCACUUGGAUUUCGUUCCCUGCU	UUGUCUCCAGAGCAUUCCA
>mmu-mir-194-1 MI0000236	AUCGGGUGUAACAGCAACUCCAUGUGGACUGUGCUCGGAUUCCAGUGGAGCUGCUGUUACUUCUGAU	CUGUGCUCGGAUU
>mmu-mir-194-2 MI0000733	GUGGCUCCCACCCUCUGUAACAGCAACUCCAUGUGGAAGUGCCCACUGGUUCCAGUGGGGCUGCUGUUAUCUGGGGUGGCGGCUAG	AGUGCCCACUGGUU
>mmu-mir-195a MI0000237	ACACCCAACUCUCCUGGCUCUAGCAGCACAGAAAUAUUGGCAUGGGGAAGUGAGUCUGCCAAUAUUGGCUGUGCUGCUCCAGGCAGGGUGGUGA	AUGGGGAAGUGAGUCUG

>mmu-mir-200c MI0000694	CCCUCGUCUUACCCAGCAGUGUUUGGGUGCUGGUUGGGAGUCUCUAAUACUGCCGGGUAAUGAUGGAGG	GUGCUGGUUGGGAGUCUC
>mmu-mir-214 MI0000698	GGCCUGGCUGGACAGAGUUGUCAUGUGUCUGCCUGUCUACACUUGCUGUGCAGAACAUCCGCUCACCUGUACAGCAGGCACAGACAGGCAGUCACAUGACAACCCAGCCU	AGAACAUCCGCUCACCUGU
>mmu-mir-320 MI0000704	GCCUCGCCGCCCUCCGCCUUCUCUUCCCGGUUCUUCCCGGAGUCGGGAAAAGCUGGGUUGAGAGGGCGAAAAAGGAUGUGGG	CGGAGUCGGG
>mmu-mir-497a MI0004636	CCUGCCCCCGCCCCAGCAGCACACUGUGGUUUGUACGGCACUGUGGCCACGUCCAAACCACACUGUGGUGUUAGAGCGAGGGUA	CGGCACUGUGGCCACGUC
	Mouse-Control miRNAs	
>mmu-mir-10a MI0000685	GACCUGUCUGUCUUCUGUAUAUACCCUGUAGAUCCGAAUUUGUGUAAGGAAUUUUGUGGUCACAAAUUCGUAUCUAGGGGAAUAUGUAGUUGACAUAAACACUCCGCUCA	UAAGGAAUUUUGUGGUCA
>mmu-mir-9-1 MI0000720	CGGGGUUGGUUGUUAUCUUUGGUUAUCUAGCUGUAUGAGUGGUGUGGAGUCUUCAUAAAGCUAGAUAACCGAAAGUAAAAAUAACCCCA	GUGGUGUGGAGUCUUC
>mmu-mir-34b MI0000404	GUGCUCGGUUUGUAGGCAGUGUAAUUAGCUGAUUGUAGUGCGGUGCUGACAAUCACUAACUCCACUGCCAUCAAAACAAGGCAC	AGUGCGGUGCUGAC
>mmu-mir-92a-1 MI0000719	CUUUCUACACAGGUUGGGAUUUGUCGCAAUGCUGUGUUUCUCUGUAUGGUAUUGCACUUGUCCCGGCCUGUUGAGUUUGG	GUGUUUCUCUGUAUGG
>mmu-mir-365-2 MI0001645	AGAGUGAUCAAGGACAGCAAGAAAAAUGAGGGACUUUCAGGGGCAGCUGUGUUUCCUGACUCAGUCAUAAUGCCCCUAAAAAUCCUUAUUGUUCUUGCAGUGUGCAUCGGAG	UUUCCUGACUCAGUCA
>mmu-mir-431 MI0001524	CGUCCUGCGAGGUGUCUUGCAGGCCGUCAUGCAGGCCACACUGACGGUAACGUUGCAGGUCGUCUUGCAGGGCUUCUCGCAAGACGACAUC	GGCCACACUGACGGUAACGUUG
>mmu-mir-25 MI0000689	GGCCAGUGUUGAGAGGCGGAGACUUGGGCAAUUGCUGGACGCUGCCCUGGGCAUUGCACUUGUCUCGGUCUGACAGUGCCGGCC	UGGACGCUGCCCUGGG
>mmu-mir-28a MI0000690	GGUCCCUACCUUCAAGGAGCUCACAGUCUAUUGAGUUGCCUUUCUGAUUCUCCCACUAGAUUGUGAGCUGCUGGAGGGCAGGCACU	UUGCCUUUCUGAUUCUCC

>mmu-mir-181c MI0000724	GCCAAGGGUUUGGGGGAACAUUCAACCUGUCGGUGAGUUUGGGCAGCUCAGACAAACCAUCGACCGUUGAGUGGACCCCGAGGCCUGGA	UUGGGCAGCUCAGACAA
>mmu-mir-187 MI0000229	UCAGGCUACAACACAGGACCCGGGCGCUGCUCUGACCCCUCGUGUCUUGUGUUGCAGCCGG	CGCUGCUCUGACCCC
>mmu-mir-19b-1 MI0000718	CACUGGUCUAUGGUUAGUUUUGCAGGUUUGCAUCCAGCUGUAUAAUAUUCUGCUGUGCAAAUCCAUGCAAAACUGACUGUGGUGGUG	UGUAUAAUAUUCUGC
>mmu-mir-22 MI0000570	ACCUGGCUGAGCCGCAGUAGUUCUUCAGUGGCAAGCUUUAUGUCCUGACCCAGCUAAAGCUGCCAGUUGAAGAACUGUUGCCCUCUGCCCCUGGC	UGUCCUGACCCAGCUA
>mmu-mir-153 MI0000175	CGGUGUCAUUUUUGUGACGUUGCAGCUAGUAAUAUGAGCCCAGUUGCAUAGUCACAAAAGUGAUCAUUG	AGUAAUAUGAGCCCAG
>mmu-mir-124-1 MI0000716	AGGCCUCUCUCUCCGUGUUCACAGCGGACCUUGAUUUAAAUGUCCAUACAAUUAAGGCACGCGGUGAAUGCCAAGAAUGGGGCUG	UUAAAUGUCCAUACAAU
>mmu-mir-219a-2 MI0000741	ACUCAGGGGCUUCGCCACUGAUUGUCCAAACGCAAUUCUUGUACGAGUCUGCGGCCAACCGAGAAUUGUGGCUGGACAUCUGUGGUUGAGCUCCGGG	UGUACGAGUCUGCGGCCAACCG
>mmu-mir-202 MI0000245	GUUCCUUUUUCCUAUGCAUAUACUUCUUUGUGGAUCUGGUCUAAAGAGGUAUAGCGCAUGGGAAGAUGGAGC	GUGGAUCUGGUCUAA
>mmu-mir-216a MI0000699	UUGGUUUAAUCUCAGCUGGCAACUGUGAGAUGUCCCUAUCAUUCCUCACAGUGGUCUCUGGGAUUAUGCUAA	GAUGUCCCUAUCAUUCCU
>mmu-mir-301b MI0004122	UUUCCUGCUGGCUGCGGGUGCUCUGACUAGGUUGCACUACUGUGCUGUGAGAAGCAGUGCAAUGGUAUUGUCAAAGCAUCUGGGACCAGCCUCGAAG	GUGCUGUGAGAAG
>mmu-mir-1306 MI0009935	CAGUCUCCACCACCUCCCCUGCAAACGUCCAGUGAUGCAGAGGUAAUGGACGUUGGCUCUGGUGGUGAUGGACAGUCCG	AGUGAUGCAGAGGUAAUGG
>mmu-mir-217 MI0000731	AAACAUAGUCAUUACAGUUUUUGAUGUUGCAGAUACUGCAUCAGGAACUGACUGGAUAAGACUUAAUCCCCAUCAGUUCCUAAUGCAUUGCCUUCAGCAUCUAAACAA	UAAGACUUAAUCCC
>mmu-mir-20b MI0003536	CCUAGUAGUGCCAAAGUGCUCAUAGUGCAGGUAGUUUUUAUACCACUCUACUGCAGUGUGAGCACUUCUAGUACUCCUGG	UUUUUAUACCACUCU

>mmu-mir-93 MI0000581	AGUCAUGGGGGCUCCAAAGUGCUGUUCGUGCAGGUAGUGUAAUUACCUGACCUACUGCUGAGCUAGCACU UCCCGAGCCCCCAGGACA	UGUAAUUACCUGACCU
>mmu-mir-190a MI0000232	CUGUGUGAUAUGUUUGAUAUAUUAGGUUGUUAUUUAAUCCAACUAUAUAUCAAGCAUAUUCCUACAG	UGUUAUUUAAUCCA
>mmu-mir-196a-1 MI0000552	UGAGCCGGGACUGUUGAGUGAAGUAGGUAGUUUCAUGUUGUUGGGCCUGGCUUUCUGAACACAACGACAU CAAACCACCUGAUUCAUGGCAGUUACUGCUUC	CCUGGCUUUCUGAACA
>mmu-mir-375 MI0000792	CCCCGCGACGAGCCCCUCGCACAAACCGGACCUGAGCGUUUUGUUCGUUCGGCUCGCGUGAGGC	CGGACCUGAGCGU
>mmu-mir-222 MI0000710	CCCUCAGUGGCUCAGUAGCCAGUGUAGAUCCUGUCUUUGGUAAUCAGCAGCUACAUCUGGCUACUGGGUC UCUGGUGGC	GUCUUUGGUAAUCAGC
>mmu-mir-671 MI0004133	UGGCAGGCCAGGAAGAGGAGGAAGCCCUGGAGGGGCUGGAGGUGAUGGAUGUUUUCCUCCGGUUCUCAGG GCUCCACCUCUUUCGAGCCGUAGAGCCA	GUGAUGGAUGUUUUCC
>mmu-mir-1a-1 MI0000139	GCUUGGGACACAUACUUCUUUAUAUGCCCAUAUGAACCUGCUAAGCUAUGGAAUGUAAAGAAGUAUGUA UUUCAGGC	UGAACCUGCUAAGCUA
>mmu-mir-205 MI0000248	CUCUUGUCCUUCAUUCCACCGGAGUCUGUCUUAUGCCAACCAGAUUUCAGUGGAGUGAAGCUCAGGAG	UCUUAUGCCAACCA
>mmu-mir-300 MI0000400	GCUACUUGAAGAGAGGUUAUCCUUUGUGUGUUUGCUUUACGCGAAAUGAAUAUGCAAGGGCAAGCUCUCU UCGAGGAGC	GUGUUUGCUUUACGCGA AAUGAA
>mmu-mir-32 MI0000691	GGAGAUAUUGCACAUUACUAAGUUGCAUGUUGUCACGGCCUCAAUGCAAUUUAGUGUGUGUGAUAUUUU C	UGUUGUCACGGCCUCAA UG
>mmu-mir-127 MI0000154	CCAGCCUGCUGAAGCUCAGAGGGCUCUGAUUCAGAAAGAUCAUCGGAUCCGUCUGAGCUUGGCUGGUCGG	UCAGAAAGAUCA
>mmu-mir-134 MI0000160	AGGGUGUGUGACUGGUUGACCAGAGGGGCGUGCACUCUGUUCACCCUGUGGGCCACCUAGUCACCAACCC U	CGUGCACUCUGUUCACC
>mmu-mir-136 MI0000162	GAGGACUCCAUUUGUUUUGAUGAUGGAUUCUUAAGCUCCAUCAUCGUCUCAAAUGAGUCUUC	AUUCUUAAGCUCC

>mmu-mir-140 MI0000165	CCUGCCAGUGGUUUUACCCUAUGGUAGGUUACGUCAUGCUGUUCUACCACAGGGUAGAACCACGGACAGG	GUUACGUCAUGCUGUUC
>mmu-mir-150 MI0000172	CCCUGUCUCCCAACCCUUGUACCAGUGCUGUGCCUCAGACCCUGGUACAGGCCUGGGGGAUAGGG	CUGUGCCUCAGACC
>mmu-mir-411 MI0001163	UGGUACUUGGAGAGAUAGUAGACCGUAUAGCGUACGCUUUAUCUGUGACGUAUGUAACACGGUCCACUAA CCCUCAGUAUCA	CUUUAUCUGUGACG
>mmu-mir-3090 MI0014083	UUCCUGUCCCAGUGGUCUGGGUGGGGCCUGAGAUCCUGAAUUUCUAAUGAGCUCCCAGGUGACACCCUGA CUCACUGUGCCAGAGGA	CUGAAUUUCUAAUGAGC
>mmu-mir-5627 MI0019198	AGAGGGUGCGCCGGGCCCUGCGUCCCCGCCCUAGAGGACAGGGCUCUCCGGCGCCCCUCGU	UCCCCGCCCUAGAGG
>mmu-mir-1981 MI0009992	GUAAAGGCUGGGCUUAGACGUGGCCUUUGGGUGUGGAAUGCACUUCCGUUUGUAACCGCCAUCUAACCCU GGCCUUUGACAG	CUUUGGGUGUGGAAUGC ACUUCCGUUUGUAACCG C
>mmu-mir-875 MI0005551	UCUGUGGUACUAUACCUCAGUUUUAUCAGGUGUUCAUUAAAAUCACCUGAAAAUACUGAGGCUAUGUUUC ACUGAGCA	UUCAUUAAAAUCA
>mmu-mir-675 MI0004123	UGCGGCCCAGGGACUGGUGCGGAAAGGGCCCACAGUGGACUUGGUACACUGUAUGCCCUAACCGCUCAGU CCCUGGGUCUGGCA	GGACUUGGUACA
>mmu-mir-466d MI0005546	CAUGUGUGUUUGUGUGUGCGUACAUGUACAUGUGUGUAUAUGAAUAUACAUAUACAUACACGCACACAU AGAUACGCACGCACACACACACACAGG	UGUGUGUAUAUGAAUAU ACA
>mmu-mir-351 MI0000643	CAUGGCACCUCCGUUUCCCUGAGGAGCCCUUUGAGCCUGGAGUGAAAAAAAAAAACAGGUCAAGAGGCGC CUGGGAACUGGAGAAGAGUGUUAAACUUC	GAGUGAAAAAAAAAAAC A
>mmu-mir-330 MI0000607	GACCCUUUGGCGAUCUCUGCCUCUCUGGGCCUGUGUCUUAGGCUCUUCAAGAUCCAACGAGCAAAGCACA GGGCCUGCAGAGAGGUAGCGCUCUGCUC	UCUUCAAGAUCCAACGA
>mmu-mir-292a MI0000390	CAGCCUGUGAUACUCAAACUGGGGGCUCUUUUGGAUUUUCAUCGGAAGAAAAGUGCCGCCAGGUUUUGAG UGUCACCGGUUG	GAUUUUCAUCGGAAGA

>mmu-mir-155 MI0000177	CUGUUAAUGCUAAUUGUGAUAGGGGUUUUGGCCUCUGACUGACUCCUACCUGUUAGCAUUAACAG	UUUGGCCUCUGA
>mmu-mir-182 MI0000224	ACCAUUUUUGGCAAUGGUAGAACUCACACCGGUAAGGUAAUGGGACCCGGUGGUUCUAGACUUGCCAACUAUGGU	GUAAGGUAAUGGGACCCG
>mmu-mir-17 MI0000687	GUCAGAAUAAUGUCAAAGUGCUUACAGUGCAGGUAGUGAUGUGUGCAUCUACUGCAGUGAGGGCACUUGUAGCAUUAUGCUGAC	UGAUGUGUGCAUCU
>mmu-mir-450a-1 MI0001653	GAGAGAUACUGAGCUGUUUUUGCGAUGUGUUCCUAAUAUGUGCUAUAAUUAUAUUGGGAACAUUUUGCAUAAAUAGCUUUGUGUCAAUACA	GUGCUAUAAUUAU
	Human MCF-7 cells-Estrogen regulated miRNAs (Maillot et al. 2009)	
>hsa-let-7a-1 MI0000060	UGGGAUGAGGUAGUAGGUUGUAUAGUUUUAGGGUCACACCCACCACUGGGAGAUAACUAUACAAUCUACUGUCUUUCCUA	UUAGGGUCACACCCACCACUGGGAGAUAA
>hsa-let-7a-2 MI0000061	AGGUUGAGGUAGUAGGUUGUAUAGUUUAGAAUUACAUCAAGGGAGAUAACUGUACAGCCUCCUAGCUUUCCU	UAGAAUUACAUCAAGGGAGAUAA
>hsa-let-7a-3 MI0000062	GGGUGAGGUAGUAGGUUGUAUAGUUUGGGGCUCUGCCCUGCUAUGGGAUAACUAUACAAUCUACUGUCUUUCCU	UGGGGCUCUGCCCUGCUAUGGGAUAA
>hsa-let-7c MI0000064	GCAUCCGGGUUGAGGUAGUAGGUUGUAUGGUUUAGAGUUACACCCUGGGAGUUAACUGUACAACCUUCUAGCUUUCCUUGGAGC	UAGAGUUACACCCUGGGAGUUAA
>hsa-let-7f-1 MI0000067	UCAGAGUGAGGUAGUAGAUUGUAUAGUUGUGGGGUAGUGAUUUUACCCUGUUCAGGAGAUAACUAUACAAUCUAUUGCCUUCCCUGA	GUGGGGUAGUGAUUUUACCCUGUUCAGGAGAUAA
>hsa-let-7f-2 MI0000068	UGUGGGAUGAGGUAGUAGAUUGUAUAGUUUUAGGGUCAUACCCCAUCUUGGAGAUAACUAUACAGUCUACUGUCUUUCCCACG	UUAGGGUCAUACCCCAUCUUGGAGAUAA
>hsa-let-7g MI0000433	AGGCUGAGGUAGUAGUUUGUACAGUUUGAGGGUCUAUGAUACCACCCGGUACAGGAGAUAACUGUACAGGCCACUGCCUUGCCA	UGAGGGUCUAUGAUACCACCCGGUACAGGAGAUAA
>hsa-mir-21 MI0000077	UGUCGGGUAGCUUAUCAGACUGAUGUUGACUGUUGAAUCUCAUGGCAACACCAGUCGAUGGGCUGUCUGACA	CUGUUGAAUCUCAUGG
>hsa-mir-23a MI0000079	GGCCGGCUGGGGUUCCUGGGGAUGGGAUUUGCUUCCUGUCACAAAUCACAUUGCCAGGGAUUUCCAACCGACC	GCUUCCUGUCACAA
>hsa-mir-23b MI0000439	CUCAGGUGCUCUGGCUGCUUGGGUUCCUGGCAUGCUGAUUUGUGACUUAAGAUUAAAAUCACAUUGCCAGGGAUUACCACGCAACCACGACCUUGGC	GUGACUUAAGAUUAAA
>hsa-mir-24-1 MI0000080	CUCCGGUGCCUACUGAGCUGAUAUCAGUUCUCAUUUUACACACUGGCUCAGUUCAGCAGGAACAGGAG	UCUCAUUUUACACAC
>hsa-mir-24-2 MI0000081	CUCUGCCUCCCGUGCCUACUGAGCUGAAACACAGUUGGUUUGUGUACACUGGCUCAGUUCAGCAGGAACAGGG	UUGGU

>hsa-mir-26a-1 MI0000083	GUGGCCUCGUUCAAGUAAUCCAGGAUAGGCUGUGCAGGUCCCAAUGGGCCUAUUCUUGGUUACUUGCAC GGGGACGC	GUGCAGGUCCCAAUGGG
>hsa-mir-26a-2 MI0000750	GGCUGUGGCUGGAUUCAAGUAAUCCAGGAUAGGCUGUUUCCAUCUGUGAGGCCUAUUCUUGAUUACUUG UUUCUGGAGGCAGCU	GUUUCCAUCUGUGAGG
>hsa-mir-26b MI0000084	CCGGGACCCAGUUCAAGUAAUUCAGGAUAGGUUGUGUGCUGUCCAGCCUGUUCUCCAUUACUUGGCUCG GGGACCGG	UGUGUGCUGUCCAG
>hsa-mir-27a MI0000085	CUGAGGAGCAGGGCUUAGCUGCUUGUGAGCAGGGUCCACACCAAGUCGUGUUCACAGUGGCUAAGUUCC GCCCCCCAG	GGGUCCACACCAAGUCGU G
>hsa-mir-27b MI0000440	ACCUCUCUAACAAGGUGCAGAGCUUAGCUGAUUGGUGAACAGUGAUUGGUUUCCGCUUUGUUCACAGUG GCUAAGUUCUGCACCUGAAGAGAAGGUG	AGUGAUUGGUUUCCGCUU UG
>hsa-mir-98 MI0000100	AGGAUUCUGCUCAUGCCAGGGUGAGGUAGUAAGUUGUAUUGUUGUGGGGUAGGGAUAUUAGGCCCCAAU UAGAAGAUAACUAUACAACUUACUACUUUCCCUGGUGUGUGGCAUAUUCA	GUGGGGUAGGGAUAUUAG GCCCCAAUUAGAAGAUAA
>hsa-mir-181a-1 MI0000289	UGAGUUUUGAGGUUGCUUCAGUGAACAUUCAACGCUGUCGGUGAGUUUGGAAUUAAAAUCAAAACCAUC GACCGUUGAUUGUACCCUAUGGCUAACCAUCAUCUACUCCA	UUGGAAUUAAAAUCAAA
>hsa-mir-181a-2 MI0000269	AGAAGGGCUAUCAGGCCAGCCUUCAGAGGACUCCAAGGAACAUUCAACGCUGUCGGUGAGUUUGGGAUU UGAAAAAACCACUGACCGUUGACUGUACCUUGGGGUCCUUA	UUGGGAUUUGAAAAA
>hsa-mir-181b-1 MI0000270	CCUGUGCAGAGAUUAUUUUUUAAAAGGUCACAAUCAACAUUCAUUGCUGUCGGUGGGUUGAACUGUGUG GACAAGCUCACUGAACAAUGAAUGCAACUGUGGCCCCGCUU	UGAACUGUGUGGACAAG
>hsa-mir-181b-2 MI0000683	CUGAUGGCUGCACUCAACAUUCAUUGCUGUCGGUGGGUUUGAGUCUGAAUCAACUCACUGAUCAAUGAA UGCAAACUGCGGACCAAACA	UUGAGUCUGAAUCAA
>hsa-mir-181d MI0003139	GUCCCCUCCCCUAGGCCACAGCCGAGGUCACAAUCAACAUUCAUUGUUGUCGGUGGGUUGUGAGGACUG AGGCCAGACCCACCGGGGGAUGAAUGUCACUGUGGCUGGGCCAGACACGGCUUAAGGGGAAUGGGGAC	UGUGAGGACUGAGGCCAG AC
>hsa-mir-193a MI0000487	CGAGGAUGGGAGCUGAGGGCUGGGUCUUUGCGGGCGAGAUGAGGGUGUCGGAUCAACUGGCCUACAAAG UCCCAGUUCUCGGCCCCCG	GGGUGUCGGAUC
>hsa-mir-193b MI0003137	GUGGUCUCAGAAUCGGGGUUUUGAGGGCGAGAUGAGUUUAUGUUUUAUCCAACUGGCCCUCAAAGUCCC GCUUUUGGGGUCAU	GUUUAUGUUUUAUCC
>hsa-mir-200a MI0000737	CCGGGCCCCUGUGAGCAUCUUACCGGACAGUGCUGGAUUUCCCAGCUUGACUCUAACACUGUCUGGUAAC GAUGUUCAAAGGUGACCCGC	UUUCCCAGCUUGACUC
>hsa-mir-200c MI0000650	CCCUCGUCUUACCCAGCAGUGUUUGGGUGCGGUUGGGAGUCUCUAAUACUGCCGGGUAAUGAUGGAGG	GUGCGGUUGGGAGUCUC

>hsa-mir-203a MI0000283	GUGUUGGGGACUCGCGCGCUGGGUCCAGUGGUUCUUAACAGUUCAACAGUUCUGUAGCGCAAUUGUGAA AUGUUUAGGACCACUAGACCCGGCGGGCGCGGCGACAGCGA	CUGUAGCGCAAUU
>hsa-mir-203b MI0017343	GCGCCCGCCGGGUCUAGUGGUCCUAAACAUUUCACAAUUGCGCUACAGAACUGUUGAACUGUUAAGAAC CACUGGACCCAGCGCGC	AUUGCGCUACAGAACUG
>hsa-mir-499a MI0003183	GCCCUGUCCCCUGUGCCUUGGGCGGGCGGCUGUUAAGACUUGCAGUGAUGUUUAACUCCUCUCCACGUGA ACAUCACAGCAAGUCUGUGCUGCUUCCCGUCCCUACGCUGCCUGGGCAGGGU	AACUCCUCUCCACGUG
>hsa-mir-499b MI0017396	GGAAGCAGCACAGACUUGCUGUGAUGUUCACGUGGAGAGGAGUUAAACAUCACUGCAAGUCUUAACAGC CGCC	CGUGGAGAGGAGUUA
>hsa-mir-520d* MI0003164	UCUCAAGCUGUGAGUCUACAAAGGGAAGCCCUUUCUGUUGUCUAAAAGAAAAGAAAGUGCUUCUCUUUG GUGGGUUACGGUUUGAGA	UGUUGUCUAAAAG
	Human-Control miRNAs	
>hsa-mir-10a MI0000266	GAUCUGUCUGUCUUCUGUAUAUACCCUGUAGAUCCGAAUUUGUGUAAGGAAUUUUGUGGUCACAAAUUC GUAUCUAGGGGAAUAUGUAGUUGACAUAAACACUCCGCUCU	UAAGGAAUUUUGUGGUCA
>hsa-mir-9-1 MI0000466	CGGGGUUGGUUGUUAUCUUUGGUUAUCUAGCUGUAUGAGUGGUGUGGAGUCUUCAUAAAGCUAGAUAA CCGAAAGUAAAAAUAACCCCA	GUGGUGUGGAGUCUUC
>hsa-mir-34b MI0000742	GUGCUCGGUUUGUAGGCAGUGUCAUUAGCUGAUUGUACUGUGGUGGUUACAAUCACUAACUCCACUGCC AUCAAAACAAGGCAC	UACUGUGGUGGUUA
>hsa-mir-92a-1 MI0000093	CUUUCUACACAGGUUGGGAUCGGUUGCAAUGCUGUGUUUCUGUAUGGUAUUGCACUUGUCCCGGCCUGU UGAGUUUGG	GUGUUUCUGUAUGG
>hsa-mir-365b MI0000769	AGAGUGUUCAAGGACAGCAAGAAAAAUGAGGGACUUUCAGGGGCAGCUGUGUUUUCUGACUCAGUCAUA AUGCCCCUAAAAAUCCUUAUUGUUCUUGCAGUGUGCAUCGGG	GUUUUCUGACUCAGUCA
>hsa-mir-431 MI0001721	UCCUGCUUGUCCUGCGAGGUGUCUUGCAGGCCGUCAUGCAGGCCACACUGACGGUAACGUUGCAGGUCGU CUUGCAGGGCUUCUCGCAAGACGACAUCCUCAUCACCAACGACG	GGCCACACUGACGGUAAC GUUG
>hsa-mir-25 MI0000082	GGCCAGUGUUGAGAGGCGGAGACUUGGGCAAUUGCUGGACGCUGCCCUGGGCAUUGCACUUGUCUCGGU CUGACAGUGCCGGCC	CUGGACGCUGCCCUGGG
>hsa-mir-28 MI0000086	GGUCCUUGCCCUCAAGGAGCUCACAGUCUAUUGAGUUACCUUUCUGACUUUCCCACUAGAUUGUGAGCU CCUGGAGGGCAGGCACU	UUACCUUUCUGACUUUCC

>hsa-mir-187 MI0000274	GGUCGGGCUCACCAUGACACAGUGUGAGACCUCGGGCUACAACACAGGACCCGGGCGCUGCUCUGACCCC UCGUGUCUUGUGUUGCAGCCGGAGGGACGCAGGUCCGCA	GCUGCUCUGACCCC
>hsa-mir-19b-1 MI0000074	CACUGUUCUAUGGUUAGUUUUGCAGGUUUGCAUCCAGCUGUGUGAUAUUCUGCUGUGCAAAUCCAUGCA AAACUGACUGUGGUAGUG	UGUGUGAUAUUCUGC
>hsa-mir-22 MI0000078	GGCUGAGCCGCAGUAGUUCUUCAGUGGCAAGCUUUAUGUCCUGACCCAGCUAAAGCUGCCAGUUGAAGA ACUGUUGCCCUCUGCC	UGUCCUGACCCAGCUA
>hsa-mir-153-2 MI0000464	AGCGGUGGCCAGUGUCAUUUUUGUGAUGUUGCAGCUAGUAAUAUGAGCCCAGUUGCAUAGUCACAAAAG UGAUCAUUGGAAACUGUG	AGUAAUAUGAGCCCAG
>hsa-mir-124-1 MI0000443	AGGCCUCUCUCUCCGUGUUCACAGCGGACCUUGAUUUAAAUGUCCAUACAAUUAAGGCACGCGGUGAAU GCCAAGAAUGGGGCUG	UUAAAUGUCCAUACAAU
>hsa-mir-219a-1 MI0000296	CCGCCCCGGGCCGCGGCUCCUGAUUGUCCAAACGCAAUUCUCGAGUCUAUGGCUCCGGCCGAGAGUUGAG UCUGGACGUCCCGAGCCGCCGCCCCCAAACCUCGAGCGGG	CGAGUCUAUGGCUCCGGC CG
>hsa-mir-202 MI0003130	CGCCUCAGAGCCGCCCGCCGUUCCUUUUUCCUAUGCAUAUACUUCUUUGAGGAUCUGGCCUAAAGAGGUA UAGGGCAUGGGAAAACGGGGCGGUCGGGUCCUCCCCAGCG	AGGAUCUGGCCUAA
>hsa-mir-216a MI0000292	GAUGGCUGUGAGUUGGCUUAAUCUCAGCUGGCAACUGUGAGAUGUUCAUACAAUCCCUCACAGUGGUCU CUGGGAUUAUGCUAAACAGAGCAAUUUCCUAGCCCUCACGA	GAUGUUCAUACAAUCCC
>hsa-mir-301b MI0005568	GCCGCAGGUGCUCUGACGAGGUUGCACUACUGUGCUCUGAGAAGCAGUGCAAUGAUAUUGUCAAAGCAU CUGGGACCA	GUGCUCUGAGAAG
>hsa-mir-1306 MI0006443	GUGAGCAGUCUCCACCACCUCCCCUGCAAACGUCCAGUGGUGCAGAGGUAAUGGACGUUGGCUCUGGUGG UGAUGGACAGUCCGA	GUGGUGCAGAGGUAAUGG
>hsa-mir-20b MI0001519	AGUACCAAAGUGCUCAUAGUGCAGGUAGUUUUGGCAUGACUCUACUGUAGUAUGGGCACUUCCAGUACU	UUUUGGCAUGACUCU
>hsa-mir-93 MI0000095	CUGGGGGCUCCAAAGUGCUGUUCGUGCAGGUAGUGUGAUUACCCAACCUACUGCUGAGCUAGCACUUCC CGAGCCCCCGG	UGUGAUUACCCAACCU
>hsa-mir-190a MI0000486	UGCAGGCCUCUGUGUGAUAUGUUUGAUAUAUUAGGUUGUUAUUUAAUCCAACUAUAUAUCAAACAUAU UCCUACAGUGUCUUGCC	UGUUAUUUAAUCCAA
>hsa-mir-196a-2 MI0000279	UGCUCGCUCAGCUGAUCUGUGGCUUAGGUAGUUUCAUGUUGUUGGGAUUGAGUUUUGAACUCGGCAACA AGAAACUGCCUGAGUUACAUCAGUCGGUUUUCGUCGAGGGC	AUUGAGUUUUGAACU
>hsa-mir-222 MI0000299	GCUGCUGGAAGGUGUAGGUACCCUCAAUGGCUCAGUAGCCAGUGUAGAUCCUGUCUUUCGUAAUCAGCA GCUACAUCUGGCUACUGGGUCUCUGAUGGCAUCUUCUAGCU	GUCUUUCGUAAUCAGC

>hsa-mir-671 MI0003760	GCAGGUGAACUGGCAGGCCAGGAAGAGGAGGAAGCCCUGGAGGGGCUGGAGGUGAUGGAUGUUUUCCUCCGGUUCUCAGGGCUCCACCUCUUUCGGGCCGUAGAGCCAGGGCUGGUGC	GUGAUGGAUGUUUUCC
>hsa-mir-1-1 MI0000651	UGGGAAACAUACUUCUUUAUAUGCCCAUAUGGACCUGCUAAGCUAUGGAAUGUAAAGAAGUAUGUAUCUCA	AUGGACCUGCUA
>hsa-mir-205 MI0000285	AAAGAUCCUCAGACAAUCCAUGUGCUUCUCUUGUCCUUCAUUCCACCGGAGUCUGUCUCAUACCCAACCAGAUUUCAGUGGAGUGAAGUUCAGGAGGCAUGGAGCUGACA	UCUCAUACCCAACCA
>hsa-mir-32 MI0000090	GGAGAUAUUGCACAUUACUAAGUUGCAUGUUGUCACGGCCUCAAUGCAAUUUAGUGUGUGUGAUAUUUUC	UGUUGUCACGGCCUCAAUG
>hsa-mir-127 MI0000472	UGUGAUCACUGUCUCCAGCCUGCUGAAGCUCAGAGGGCUCUGAUUCAGAAAGAUCAUCGGAUCCGUCUGAGCUUGGCUGGUCGGAAGUCUCAUCAUC	UCAGAAAGAUCA
>hsa-mir-134 MI0000474	CAGGGUGUGUGACUGGUUGACCAGAGGGGCAUGCACUGUGUUCACCCUGUGGGCCACCUAGUCACCAACCCUC	CAUGCACUGUGUUCAC
>hsa-mir-136 MI0000475	UGAGCCCUCGGAGGACUCCAUUUGUUUUGAUGAUGGAUUCUUAUGCUCCAUCAUCGUCUCAAAUGAGUCUUCAGAGGGUUCU	UUCUUAUGCUC
>hsa-mir-140 MI0000456	UGUGUCUCUCUCUGUGUCCUGCCAGUGGUUUUACCCUAUGGUAGGUUACGUCAUGCUGUUCUACCACAGGGUAGAACCACGGACAGGAUACCGGGGCACC	GUUACGUCAUGCUGUUC
>hsa-mir-150 MI0000479	CUCCCCAUGGCCCUGUCUCCCAACCCUUGUACCAGUGCUGGGCUCAGACCCUGGUACAGGCCUGGGGGACAGGGACCUGGGGAC	CUGGGCUCAGACC
>hsa-mir-411 MI0003675	UGGUACUUGGAGAGAUAGUAGACCGUAUAGCGUACGCUUUAUCUGUGACGUAUGUAACACGGUCCACUAACCCUCAGUAUCAAAUCCAUCCCCGAG	CUUUAUCUGUGACG
>hsa-mir-3074 MI0014181	GCUCGACUCCUGUUCCUGCUGAACUGAGCCAGUGUGUAAAAUGAGAACUGAUAUCAGCUCAGUAGGCACCGGAGGGCGGGU	UGUGUAAAAUGAGAACU
>hsa-mir-5591 MI0019151	UGGGAGCUAAGCUAUGGGUAUACUGAGCUUAUGUAUGCAUCUGCAUACCCAUAGCUUAGCUCCCA	ACUGAGCUUAUGUAUGCAUCUGC
>hsa-mir-1914 MI0008335	CGUGUGAGCCCGCCCUGUGCCCGGCCCACUUCUGCUUCCUCUUAGCGCAGGAGGGGUCCCGCACUGGGAGGGGCCCUCAC	CUUCCUCUUAGCGCA
>hsa-mir-875 MI0005541	UUAGUGGUACUAUACCUCAGUUUUAUCAGGUGUUCUUAAAAUCACCUGGAAACACUGAGGUUGUGUCUCACUGAAC	UUCUUAAAAUCA
>hsa-mir-675 MI0005416	CCCAGGGUCUGGUGCGGAGAGGGCCCACAGUGGACUUGGUGACGCUGUAUGCCCUCACCGCUCAGCCCCUGGG	GACUUGGUGACG

>hsa-mir-483 MI0002467	GAGGGGGAAGACGGGAGGAAAGAAGGGAGUGGUUCCAUCACGCCUCCUCACUCCUCUCCUCCCGUCUUCU CCUCUC	UGGUUCCAUCACGCCUCC
>hsa-mir-361 MI0000760	GGAGCUUAUCAGAAUCUCCAGGGGUACUUUAUAAUUUCAAAAAGUCCCCCAGGUGUGAUUCUGAUUUGC UUC	UUUAUAAUUUCAAAAAG
>hsa-mir-330 MI0000803	CUUUGGCGAUCACUGCCUCUCUGGGCCUGUGUCUUAGGCUCUGCAAGAUCAACCGAGCAAAGCACACGGC CUGCAGAGAGGCAGCGCUCUGCCC	UCUGCAAGAUCAACCGA
>hsa-mir-296 MI0000747	AGGACCCUUCCAGAGGGCCCCCCCUCAAUCCUGUUGUGCCUAAUUCAGAGGGUUGGGUGGAGGCUCUCCU GAAGGGCUCU	UGUGCCUAAUUCA
>hsa-mir-155 MI0000681	CUGUUAAUGCUAAUCGUGAUAGGGGUUUUUGCCUCCAACUGACUCCUACAUAUUAGCAUUAACAG	UUUUGCCUCCAACUGA
>hsa-mir-182 MI0000272	GAGCUGCUUGCCUCCCCCCGUUUUUGGCAAUGGUAGAACUCACACUGGUGAGGUAACAGGAUCCGGUGG UUCUAGACUUGCCAACUAUGGGGCGAGGACUCAGCCGGCAC	GGUGAGGUAACAGGAUCC GG
>hsa-mir-17 MI0000071	GUCAGAAUAAUGUCAAAGUGCUUACAGUGCAGGUAGUGAUAUGUGCAUCUACUGCAGUGAAGGCACUUG UAGCAUUAUGGUGAC	UGAUAUGUGCAUCU
>hsa-mir-450a-1 MI0001652	AAACGAUACUAAACUGUUUUUGCGAUGUGUUCCUAAUAUGCACUAUAAAUAUAUUGGGAACAUUUUGC AUGUAUAGUUUUGUAUCAAUAUA	GCACUAUAAAUAU
>hsa-mir-454 MI0003820	UCUGUUUAUCACCAGAUCCUAGAACCCUAUCAAUAUUGUCUCUGCUGUGUAAAUAGUUCUGAGUAGUGC AAUAUUGCUUAUAGGGUUUUGGUGUUUGGAAAGAACAAUGGGCAGG	UGUGUAAAUAGUUCUGAG
>hsa-mir-508 MI0003195	CCACCUUCAGCUGAGUGUAGUGCCCUACUCCAGAGGGCGUCACUCAUGUAAACUAAAACAUGAUUGUAG CCUUUUGGAGUAGAGUAAUACACAUCACGUAACGCAUAUUUGGUGG	UAAACUAAAACA
>hsa-mir-532 MI0003205	CGACUUGCUUUCUCUCCUCCAUGCCUUGAGUGUAGGACCGUUGGCAUCUUAAUUACCCUCCCACACCCAA GGCUUGCAGAAGAGCGAGCCU	UGGCAUCUUAAUUAC
>hsa-mir-766 MI0003836	GCAUCCUCAGGACCUGGGCUUGGGUGGUAGGAGGAAUUGGUGCUGGUCUUUCAUUUUGGAUUUGACUCC AGCCCCACAGCCUCAGCCACCCCAGCCAAUUGUCAUAGGAGC	UCAUUUUGGAUUUG

Table S3. Lists of common differentially expressed genes to E2-treated zebrafish and breast cancer. Presented are fold ratios results of gene expression microarrays of zebrafish livers after treatment with E2 for12, 24 and 48 hrs, and of breast cancer vs. normal breast.

Gene	Zebrafish-E2 treatment (12 vs. 4 hrs)	Human breast (cancer vs. Normal)	Gene	Zebrafish-E2 treatment (24 vs. 4 hrs)	Human breast (cancer vs. Normal)	Gene	Zebrafish-E2 treatment (48 vs. 4 hrs)	Human breast (cancer vs. Normal)
foxo1	-3.81543	-3.286984681	cebpd	-4.39698	-3.715873006	krt15	-2.61975	-9.799437967

mgp	2.80279	-2.390571296	dusp1	-2.23362	-3.658357673	aldh1a3	1.81365	-3.831731219
hnrnpa0	-2.12121	-1.69209092	per2	-5.61861	-3.289145222	cebpd	-6.55645	-3.715873006
prim1	10.037	1.417340798	foxo1	-7.21616	-3.286984681	per2	-6.77169	-3.289145222
ero1l	2.42642	1.432254841	zfp36l2	-3.64205	-2.990533927	foxo1	-5.26883	-3.286984681
rnaseh2a	3.00748	1.433627632	f10	-1.98831	-2.906108159	per1	3.93669	-3.197253939
asf1b	18.5255	1.454146297	slc27a6	-3.83008	-2.815101002	zfp36l2	-4.81279	-2.990533927
pola2	7.74327	1.459987281	igf1	-6.27184	-2.664885124	f10	-2.89311	-2.906108159
rrm1	6.08966	1.489958926	dusp6	-2.07292	-2.596607569	csrp1	-2.18495	-2.901962759
mthfd2	-3.38525	1.504245035	tspan7	-2.69697	-2.573601486	slc27a6	-8.64909	-2.815101002
ggps1	-1.89698	1.513723548	mgp	2.17853	-2.390571296	igf1	-9.73548	-2.664885124
rfc5	3.47921	1.514018734	cyyr1	3.26514	-2.043111289	txnip	2.79376	-2.655372076
pold1	6.78738	1.548995908	ptgds	-3.02223	-1.863103863	epas1	-3.00885	-2.621489869
sec23b	-2.97717	1.648495345	cfl2	2.65817	-1.737153377	tfpi2	-3.75159	-2.429668803
smc4	6.98819	1.70670637	hnrnpa0	-2.92791	-1.69209092	mgp	2.49668	-2.390571296
dtl	14.662	1.835357884	dgat2	-3.51216	-1.670901971	rnf128	-2.62835	-2.325877835
mybl2	23.8424	1.844821634	add3	-2.28626	-1.586622115	cugbp2	-3.20665	-2.287741676
smc2	10.9393	1.899810545	mpzl2	-1.95628	-1.58179144	wee1	2.36895	-2.268617377
wdsof1	1.95377	1.908540251	mylip	-3.3032	-1.522541776	aspa	4.34759	-2.164373857
orc6l	39.7162	1.996606079	rps4x	1.70553	-1.519380517	smarca2	-2.44616	-2.143116006
pcna	7.95076	2.024529832	fstl1	33.4639	-1.51051838	sgce	-2.06081	-2.113035139
cenpk	14.0709	2.049555097	cat	-3.2306	-1.509138661	cav2	-3.72884	-2.090702147
mcm4	17.6252	2.098250835	tead1	-2.03245	-1.501055976	serhl	6.37268	-2.009605721
rmi1	5.89567	2.324631182	eif1b	-1.70681	-1.471538723	st5	-2.25223	-1.928018968
tdo2	-8.56957	2.471779729	eef1b2	2.09271	-1.450967025	thra	-2.67027	-1.906318117
tyms	36.547	3.496174985	tfdp1	2.12983	1.414310599	ccnl1	-1.78391	-1.873146955
uhrf1	16.7852	4.555218501	ilf2	2.48423	1.414319174	ptgds	-4.05337	-1.863103863
pbk	12.9965	5.151937534	xrcc4	4.19725	1.415014875	tppp3	-3.25671	-1.823331217
			prim1	24.7691	1.417340798	POLR2A	-2.48781	-1.794616293
			pigf	2.15886	1.422719719	fbxo32	-5.59889	-1.790637489
			mapre1	2.28514	1.423686224	pik3r1	1.88741	-1.781297119
			slc37a2	-2.19438	1.428426492	zfp36l1	-7.93313	-1.754658906
			mrpl35	1.77788	1.428977723	bhlhb2	2.56887	-1.720101877
			fam136a	2.51099	1.431168332	il4r	-9.25584	-1.711371363

ero1l	11.0466	1.432254841	asph	2.54849	-1.707484483
rnaseh2a	3.64306	1.433627632	hnrnpa0	-3.01253	-1.69209092
nudt1	4.27212	1.435274704	dgat2	-3.35212	-1.670901971
pgd	2.35249	1.438710194	sf3a2	1.98431	-1.616915196
sub1	2.12331	1.442626183	add3	-1.98257	-1.586622115
txnrd1	1.72042	1.44284304	ypel5	-1.72095	-1.560853046
nras	3.44101	1.444631855	eef1g	1.9011	-1.546805605
psmd1	2.74486	1.445324247	fstl1	17.6834	-1.51051838
psmc6	2.79967	1.447146633	cat	-4.3411	-1.509138661
tbl2	3.0555	1.452537134	wtap	-1.92222	-1.502643969
asf1b	35.5925	1.454146297	dio2	-38.9144	-1.468795942
ppil1	2.37249	1.454969076	gnmt	-5.51585	-1.459169814
usp5	1.84483	1.458189181	flt1	-2.12765	-1.450368894
pmm2	5.87116	1.458738209	tfdp1	1.92668	1.414310599
alg6	2.02018	1.458773155	ilf2	3.23788	1.414319174
pola2	14.1718	1.459987281	xrcc4	3.0535	1.415014875
gapdh	-2.45543	1.463665172	ephx1	7.6625	1.41553214
timp2	-5.6871	1.467501867	prim1	19.6215	1.417340798
cse1l	2.17358	1.467578674	pigf	2.83157	1.422719719
edem1	1.76062	1.467959489	mapre1	2.14555	1.423686224
psmb2	1.74832	1.471121299	nup155	4.15803	1.427710528
cd2bp2	3.77782	1.482307111	slc37a2	-1.90142	1.428426492
Slbp	14.7099	1.48531021	mrpl35	1.96658	1.428977723
nutf2	8.85058	1.485689463	fam136a	1.78568	1.431168332
slc35b1	2.87696	1.486307377	ero1l	20.1057	1.432254841
mrpl14	2.38429	1.489483513	sdhc	1.74505	1.432624661
rrm1	11.1679	1.489958926	rnaseh2a	2.27723	1.433627632
dnmt1	9.11021	1.493170264	vangl1	-2.81137	1.43613676
eif5	1.73516	1.494305035	tmed3	3.18595	1.438582331
kif11	11.6072	1.499763759	cd9	-2.31546	1.439563747
pgrmc2	2.78977	1.503000307	cant1	-2.89341	1.440125404
rfc5	10.5553	1.514018734	srpk1	-1.89795	1.441570681
hm13	2.03241	1.523191788	sub1	2.8413	1.442626183

rer1	1.80009	1.527240002	nme4	-3.76738	1.443574654
stam	1.72167	1.529470932	nras	6.31616	1.444631855
stip1	2.15636	1.532837137	psmd1	3.38609	1.445324247
slc30a5	2.05287	1.54749931	psmc6	2.76992	1.447146633
pold1	21.7502	1.548995908	fkbp4	-1.76363	1.449729834
tmco1	2.71463	1.569276615	stk38l	-1.91245	1.45103741
sec61g	2.89537	1.574475761	mastl	12.5682	1.451540388
cct5	2.30011	1.582603358	tbl2	2.57236	1.452537134
snrpb	2.13288	1.586326443	asf1b	24.3983	1.454146297
ipo9	2.27617	1.587401052	ppil1	3.14452	1.454969076
iars	2.3444	1.589895408	usp5	2.26843	1.458189181
sgpl1	-2.12949	1.592093585	pmm2	7.9622	1.458738209
atm	1.80909	1.601355475	alg6	3.5197	1.458773155
tmem49	2.23668	1.608868085	pola2	9.25999	1.459987281
psmd2	2.13579	1.611279958	gapdh	-4.92895	1.463665172
copb2	2.03515	1.629230105	tmem33	-1.86198	1.464835732
uap1	2.47554	1.63150354	cse1l	2.77585	1.467578674
p4ha2	3.20901	1.646940773	edem1	1.91663	1.467959489
psma5	2.44957	1.664836263	psmb2	1.85704	1.471121299
cct3	2.7086	1.676586786	cd2bp2	4.97323	1.482307111
atic	3.87609	1.681138854	Slbp	9.25509	1.48531021
nlk	-2.42299	1.687963062	nutf2	9.77599	1.485689463
lsm4	1.97111	1.688191735	slc35b1	2.83404	1.486307377
smc4	10.6127	1.70670637	mrpl14	1.96133	1.489483513
npl	-5.86003	1.715350843	rrm1	10.4819	1.489958926
seh1l	2.21282	1.728509515	hmgb2	6.70188	1.490474444
usp14	3.28818	1.730855476	eno3	-3.80569	1.490797923
c20orf24	2.25082	1.743396856	hsh2d	-3.63634	1.49388592
psmd3	2.47586	1.743932795	pgm3	4.11477	1.496173805
nudt5	3.17445	1.758329369	kif11	125.151	1.499763759
psmc4	2.03406	1.761362944	pgrmc2	2.15459	1.503000307
idh2	1.80843	1.778205094	ppt1	-1.74453	1.507944984
arf1	-1.81248	1.790169538	rfc5	6.91719	1.514018734

adss	2.15049	1.801385325	ssx2ip	2.29336	1.519080616
lypla1	2.03039	1.808349836	hm13	4.39345	1.523191788
actn2	-1.84881	1.815948134	rer1	2.32976	1.527240002
dtl	61.2282	1.835357884	stam	3.28385	1.529470932
mybl2	66.5984	1.844821634	nnt	2.15113	1.531210069
plk4	7.01819	1.898742735	slc30a5	2.36581	1.54749931
smc2	16.3267	1.899810545	pold1	10.2219	1.548995908
wdsof1	2.10243	1.908540251	tmco1	3.38197	1.569276615
h2afx	19.437	1.92592813	sec61g	4.16278	1.574475761
tmpo	3.74855	1.978475073	cct5	2.26977	1.582603358
orc6l	113.132	1.996606079	ipo9	2.15049	1.587401052
ggh	-1.74194	2.013006708	iars	3.13612	1.589895408
pcna	15.2001	2.024529832	sgpl1	-1.80358	1.592093585
cenpk	30.1604	2.049555097	slc39a1	-2.14988	1.592672078
kpna2	15.4713	2.085126482	agt	-1.93794	1.595674299
mcm4	28.7386	2.098250835	pdia4	4.54807	1.606044615
wdr51a	8.7916	2.204609324	tmem49	1.87835	1.608868085
trip13	10.4199	2.299644226	psmd2	2.50824	1.611279958
rmi1	7.80228	2.324631182	copb2	2.20149	1.629230105
lmnb1	2.37698	2.400206245	uap1	2.61916	1.63150354
rad51	37.9369	2.53591429	bub3	4.01934	1.636390464
rad54l	20.0014	2.928812504	p4ha2	2.15144	1.646940773
ttk	26.6462	2.933502238	cdc20	119.51	1.651354897
rrm2	19.944	3.073668947	psma5	2.4536	1.664836263
cdkn3	4.12387	3.10330563	kifc1	129.498	1.670075473
tyms	107.449	3.496174985	cct3	2.01443	1.676586786
uhrf1	42.4609	4.555218501	atic	3.64924	1.681138854
fn1	-4.16217	5.072483097	smc4	26.1882	1.70670637
pbk	40.6059	5.151937534	npl	-2.47514	1.715350843
			comtd1	-3.88031	1.721312702
			rgs4	-2.55589	1.722760956
			rtcd1	-1.79768	1.726442502
			rad21	2.56552	1.727853373

seh1l	3.31866	1.728509515
usp14	3.36637	1.730855476
c20orf24	1.93973	1.743396856
psmd3	3.86722	1.743932795
nudt5	2.91624	1.758329369
psmc4	2.1999	1.761362944
idh2	1.79505	1.778205094
kif23	113.288	1.794612857
tmem165	3.86243	1.798032339
lypla1	1.8892	1.808349836
ampd3	2.4186	1.812614615
actn2	-2.22765	1.815948134
derl1	3.83111	1.819959161
dtl	20.4795	1.835357884
mybl2	90.4897	1.844821634
plk4	16.5408	1.898742735
smc2	45.2633	1.899810545
wdsof1	1.79969	1.908540251
h2afx	43.1699	1.92592813
rab2a	1.76868	1.930261142
tmpo	7.58711	1.978475073
orc6l	70.8605	1.996606079
ggh	-2.11368	2.013006708
pcna	10.3336	2.024529832
cenpk	57.3806	2.049555097
gpsm2	4.78013	2.053051799
rnpep	2.09379	2.080784818
kpna2	78.449	2.085126482
shcbp1	18.8991	2.085423614
mcm4	13.9048	2.098250835
tacc3	22.7956	2.191710961
wdr51a	53.1455	2.204609324
trip13	23.3392	2.299644226

	cfb	-6.52074	2.307960165
	hmmr	8.90855	2.310369158
	ccnb1	262.419	2.311043153
	rmi1	6.92355	2.324631182
	lmnb1	4.59828	2.400206245
	rad51	34.4706	2.53591429
	ccnb2	48.2483	2.637727172
	tk1	7.48057	2.638434781
	aspm	46.2409	2.750297633
	top2a	94.565	2.917488708
	rad54l	48.7024	2.928812504
	racgap1	20.8442	2.929986682
	ttk	206.479	2.933502238
	rrm2	41.9177	3.073668947
	cdkn3	12.2778	3.10330563
	melk	59.6723	3.325744311
	cdc2	182.604	3.336384246
	tyms	159.059	3.496174985
	dlg7	95.5377	3.652341315
	nek2	91.2569	3.926110638
	uhrf1	10.8183	4.555218501
	fn1	-5.24084	5.072483097
	pbk	220.286	5.151937534
